# Fast-growing intracellular *Mycobacterium tuberculosis* populations evade antibiotic treatment

**DOI:** 10.1038/s41467-026-75915-8

**Published:** 2026-07-28

**Authors:** Nathan J. Day, Baptiste Pradel, Chak Hon Luk, Pierre Santucci, Antony Fearns, Angela Rodgers, Beren Aylan, Laure Botella, Julien Vaubourgeix, Julio Ortiz Canseco, Natalia Athanasiadi, Maximiliano G. Gutierrez

**Affiliations:** 1 Host-Pathogen Interactions in Tuberculosis Laboratory, The Francis Crick Institute, London, United Kingdom; 2 Institut de Recherche en Santé Digestive (IRSD), Université de Toulouse, INSERM, INRAE, ENVT, UPS, Toulouse, France; 3 Present Address: Blizard Institute, Faculty of Medicine and Dentistry, Queen Mary University of London, London, United Kingdom; 4 Present Address: Aix Marseille University, CNRS, LISM, IMM FR3479, IM2B Marseille, France; 5 Present Address: Boston Children’s Hospital, Harvard Medical School, Boston, USA; 6 Present Address: Luxembourg Centre for Systems Biomedicine (LCSB), University of Luxembourg, Esch-sur-Alzette, Luxembourg

**Keywords:** Antimicrobial resistance, Cellular microbiology, Bacteria, Pathogens

## Abstract

Slow growth rates are considered a hallmark of *Mycobacterium tuberculosis* (Mtb) and have been historically associated with persistence and subsequent drug tolerance. Despite the fundamental role intracellular growth plays in the pathogenesis of tuberculosis (TB), approaches that define intracellular growth rates have remained challenging. Here, we developed a high-throughput, live-cell imaging approach to quantify Mtb replication within human macrophage populations at high spatiotemporal, single-cell resolution. Unexpectedly, we discovered fast-growing intracellular Mtb populations with doubling times below 10 hours. Importantly, when treated with first-line antibiotics, these intracellular fast-growing subpopulations remained. To validate this in vivo, we developed a mouse model of tuberculosis featuring a Mtb-Timer fluorophore that reports replication activity. Single-cell analysis revealed a strong correlation between heavily burdened host cells and Mtb populations with heightened replicative activity. These data identify fast-growing intracellular bacteria as a mechanism of antibiotic evasion and challenges the prevailing idea that attributes antibiotic tolerance to bacterial dormancy.

## Abstract (web summary)

The slow growth displayed by *Mycobacterium tuberculosis* has been historically associated with pathogen’s persistence and drug tolerance. Unexpectedly, here the authors identify a rare subpopulation of fast-growing *M. tuberculosis* bacteria within infected macrophages that evades antibiotic treatment and is associated with antibiotic failure in a mouse model of tuberculosis.

## Introduction

Tuberculosis (TB) is one of the most devastating infectious diseases throughout human history, affecting humanity for over 15,000 years^1^ and resulting in 1.23 million deaths in 2024^2^. The current chemotherapy regimen for TB requires at least four months and four antibiotics to cure the disease^3^. The reasons behind this prolonged treatment duration and multi-drug requirements are multifactorial and remain poorly understood^4–6^.

*Mycobacterium tuberculosis* (Mtb), the causative agent of TB, can adopt an intracellular lifestyle and replicate within host cells during infection^7^. In the host, Mtb faces diverse intracellular and extracellular microenvironments that results in single-cell phenotypic variability^8,9^. Compelling evidence indicates a host cell contribution to antibiotic accumulation and efficacy^10–14^, but the underlying mechanisms remain poorly defined.

Mtb is canonically defined as a slow growing bacterium, characterised by bulk population doubling times of approximately 18–24 h^15^. These slow or non-growing bacterial phenotypes are frequently associated with the ability of Mtb to evade antibiotic treatment^16–18^. Previous in cellulo studies have reported bulk intracellular Mtb doubling times of 21, 26–28, and 24.7h, respectively^12,19,20^. An in vivo study reported computationally modelled Mtb doubling times of 48 hours extracellularly and 24 hours intracellularly^21^. However, these metrics obscure significant underlying heterogeneity and only recently various single cell studies have begun to report a wide range of intracellular and extracellular replication behaviours. These single-cell approaches revealed that individual Mtb bacilli have the capacity to replicate significantly faster than the canonical average, with doubling times as short as ~17 hours in vitro or in cellulo^20,22,23^. However, how these discrete replication phenotypes can potentially impact antibiotic efficacy against intracellular Mtb remain elusive.

Antibiotic efficacy has been primarily determined using parameters derived from in vitro planktonic cultures such as minimal inhibitory and minimal bactericidal concentrations^24^ that neglect the host cell contribution towards antibiotic efficacy^25^. Single-cell image-based in vitro studies of antibiotic responses address the highly heterogenous nature of Mtb infection^26,27^, but they are biased by nutrient-rich and aerobic culture conditions. On the other hand, single-cell image-based in cellulo studies overcome bulk determinations and mimic physiological intracellular environments^20,23^, but are often limited in spatial and temporal resolutions.

To address these challenges, we developed a high-content imaging platform designed to monitor Mtb intracellular replication at the single-macrophage level. This approach combines high spatiotemporal resolution with multi-day acquisition, enabling the precise quantification of dynamic and transient single-cell doubling times. To ensure physiological relevance, we implemented this workflow in human induced pluripotent stem cell-derived macrophages (iPSDM) infected with Mtb H37Rv wild-type, a well characterised and genetically defined human cell model of infection^28,29^. We also infected iPSDM with a mutant of Mtb which lacks the region of difference 1 (RD1) genomic locus. Mtb ΔRD1 localises in membrane-bound compartments (such as the phagosome). This mutant is restricted within human macrophages and was used to validate attenuated intracellular growth^30^. We profiled the standard first-line anti-TB regimen of Pyrazinamide (PZA), Isoniazid (INH) and Rifampicin (RIF) on this model system, representing three distinct mechanisms of action: intracellular acidification and membrane energetics; cell wall synthesis and bacterial transcription, respectively^31–33^. We discovered that a subpopulation of infected human macrophages harboured exceptionally fast-growing Mtb populations. Moreover, we show that these fast-growing intracellular Mtb populations evade antibiotic treatment in cellulo. Finally, to validate these phenotypes in vivo, we used a fluorescent Mtb-Timer reporter to infect TB susceptible mice combined with single cell analysis in the lungs. We show that these growing populations remain during antibiotic treatment, likely representing a phenotypically and clinically relevant reservoir of fast-growing bacteria that evades antibiotic treatment.

## Results

### An image-based pipeline to study Mtb replication in single human macrophages with high spatiotemporal resolution yields heterogenous growth profiles

**Fig. 1:**
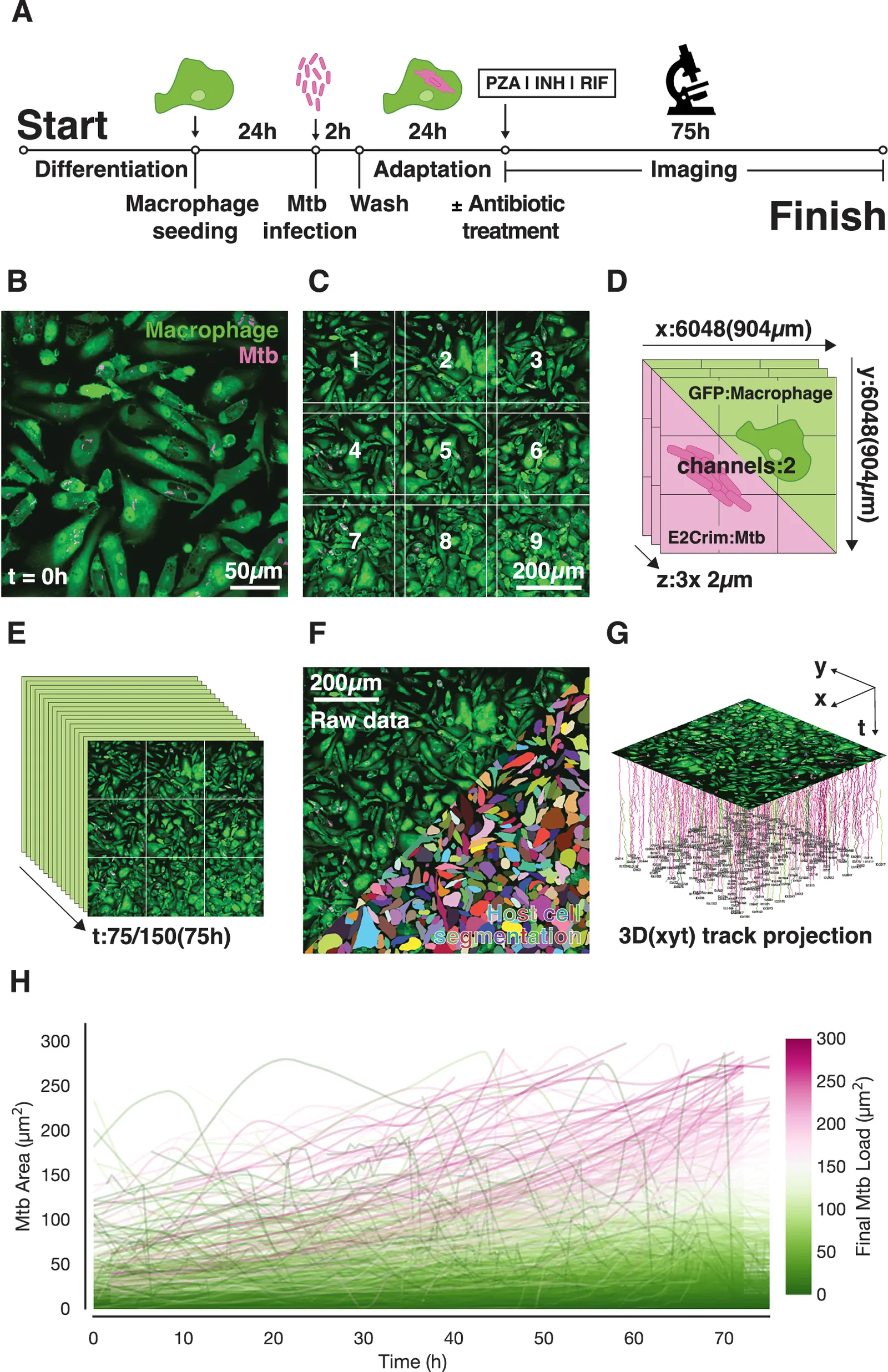
An image-based pipeline to study Mtb replication in single human macrophages with high spatiotemporal resolution yields heterogenous growth profiles. **A** Timeline of the experimental design and imaging conditions. **B** A single image acquisition, representative of *n* = 4 biological repeats, showing EGFP-expressing macrophages infected with Mtb H37Rv WT-E2-Crimson. **C** A full mosaic of 9 images with 10% overlap and single tiles shown with white square inset. **D** Each mosaic is composed of 2 fluorescent channels and 3 z-slices with a step of 2 μm. **E** The image acquisition lasts for 75 h at 30/60 min frame rates. **F** The image volume is segmented using the EGFP channel. **G** A 3D (*x, y, t*) spatiotemporal visualisation showing cell positions through time. **H** A graph showing the quantification of intracellular Mtb growth per each single-cell trajectory (*N*_*cell*_ = 3,369) in *n* = 4 biological replicates (only untreated control profiles shown), color-coded to the final intracellular Mtb burden.

We developed a high-content, live-cell imaging approach that allows for the monitoring of Mtb replication in large populations of infected macrophages (Fig. 1A). This system consists of human iPSDM expressing EGFP from an AAV locus^28^ infected with Mtb H37Rv expressing fluorescent protein E2-Crimson (Fig. 1B). Using this biological model, we established an imaging pipeline that allows for 2-channel imaging of Mtb-infected macrophages in 96-well plates, with 9 overlapping fields of view per well, across 3 × 2 µm z-slices, for up to 75 h at 30/60 min frame rates, after an initial 24 h set-up of the infection (Fig. 1C–E, Movie S1). This control assay was repeated across *n* = 4 biological replicates. In addition to this control scenario, macrophages were also treated with pre-determined EC50 and EC99 concentrations of first line antibiotics: PZA, INH and RIF, to quantify the single-cell dependency of antibiotic efficacy.

After establishing a robust image tiling, segmentation and tracking strategy (Fig. 1F, G), we were able to reliably track *N*_*cell*_ = 18,152 macrophages over a minimum period of 35 h, approximately half the total time lapse acquisition (Fig. 1H). A subsequent analysis of intracellular Mtb growth trajectories revealed a heterogeneity in Mtb replication dynamics. Some macrophage tracks showed a high intracellular Mtb load (magenta lines, Fig. 1H), whilst others harboured only low levels of Mtb intracellular populations (green lines Fig. 1H).

In these single-cell growth profiles, a subset of intracellular populations showed near instantaneous increases in intracellular Mtb burden, warranting further investigation. At the infected macrophage level, we observed three clear phenotypes related to the fast-growing Mtb: (i) Intracellular growth of Mtb (Figure S1A, Movie S3); (ii) Extracellular uptake of Mtb, where rapid increases in intracellular burden resulted in increased single cell Mtb signals that were not associated with intracellular growth (Figure S1B, uptake highlighted with a white arrow, Movie S3); and (iii) Intercellular transfer of Mtb between neighbouring macrophages, where sudden increases in intracellular burden yielded rapid growth rates not associated to intracellular replication (Figure S1C, transfer highlighted with a white arrow, Movie S3). Incidences of extrinsic Mtb growth, particularly intercellular transfer of populations of Mtb, were largely attributable to macrophage cell death^20,30^, however without a cell death marker we were unable to quantitatively investigate this effect further. To exclude the increase in intracellular signal associated with extracellular uptake and intercellular transfer in any further analysis, we defined these two processes as belonging to an “extrinsic Mtb growth” phenotype, in contrast to truly “intrinsic” intracellular Mtb growth.

In order to further assess Mtb growth dynamics, we fitted a locally weighted scatterplot smoothing (LOWESS) model to the Mtb growth trajectories^34^. This approach allowed for the accurate quantification of growth dynamics by attenuating high-frequency noise from the intracellular Mtb measurements whilst retaining the underlying biological growth pattern. It also served as a quality-control metric for tracking performance, as erroneous tracking jumps or extrinsic Mtb growth events result in a poorly fitting coefficient of determination (R^2^) between the raw Mtb data and growth curve (Figure S1B-C, shown in magnified graph ROI). Using the R^2^ coefficient of determination as a ranking metric, we classified and isolated the extrinsic growth phenotypes using a human-in-the-loop approach, wherein a set of napari^35^ key-bindings were used to iterate over and label a corpus of single-cell video glimpses. We then used these manually curated labels to segregate the single-cell growth phenotypes according to whether they were wholly intrinsic, intracellular Mtb growth or extrinsic Mtb growth (Figure S1D-H).

Of the total tracked macrophages, 57.1% (*N*_*cell*_ = 10,371) were infected, out of which 3574 macrophages exhibited no intracellular growth, 5065 macrophages exhibited minor growth and 1732 macrophages harboured doubling intracellular Mtb populations (Figure S1D). We subsequently focused our growth rate analysis to these intrinsic, intracellular doubling populations for three main reasons: firstly, the metric of doubling time is already well established within the field of microbiology and it provides us with a comparison to previous bulk level studies; secondly, calculating growth rates from minor, non-doubling intracellular growth is inherently prone to stochastic signal noise fluctuations; and thirdly, the subset populations that possessed non-doubling or no measurable growth precluded a confident assessment of whether these bacteria were arrested due to acquisition time constraints. Out of the 1732 doubling instances of intracellular Mtb growth, manual classification of intrinsic versus extrinsic growth phenotypes resulted in a final corpus of 590 doubling instances that constituted the focus of our subsequent analysis.

### Single-cell quantification of intracellular doubling times reveals fast-growing intracellular Mtb evades antibiotic treatment

**Fig. 2:**
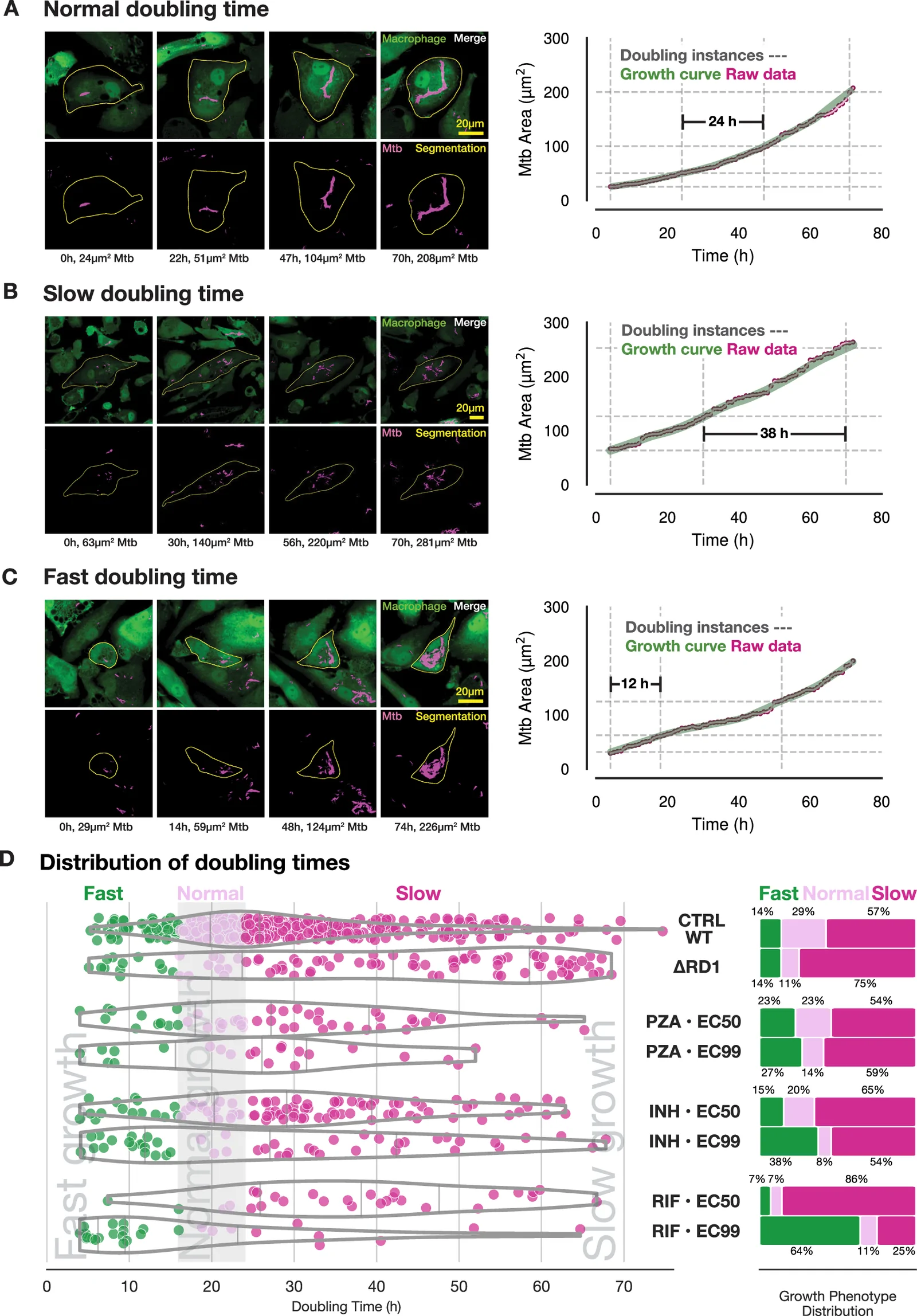
Single-cell quantification of intracellular doubling times reveals that fast-growing intracellular Mtb evades antibiotic treatment. **A** Single-cell example of an intracellular Mtb population exhibiting a “normal” doubling time (24 h). Representative cropped images (left) and LOWESS growth curve in green, raw data in magenta and the doubling points in grey dotted lines (right). **B** Single-cell example of an intracellular Mtb population exhibiting a “slow” doubling time (38 h). Representative cropped images (left) and LOWESS growth curve in green, raw data in magenta and the doubling points in grey dotted lines (right). **C** Single-cell example of an intracellular Mtb population exhibiting a “fast” doubling time (12 h). Representative cropped images (left) and LOWESS growth curve in green, raw data in magenta and the doubling points in grey dotted lines (right). **D** Distribution of intracellular doubling times attributable solely to intrinsic, intracellular Mtb growth within host-macrophages. These doubling times are separated into control (untreated) Mtb WT, control (untreated) Mtb ∆RD1 and antibiotic-treated Mtb WT, with further subdivisions according to the concentration of antibiotics (EC50 and EC99). The proportion of fast, normal and slow growth phenotypes are also exhibited as percentages for each strain of Mtb and concentration of antibiotic.

Following this, we defined three distinct phenotypes based on our observed doubling times relative to literature values: normal growth (16-24 h, Fig. 2A, Movie S2), slow growth ( ≥ 24 h, Fig. 2B, Movie S2) and fast growth ( ≤ 16 h, Fig. 2C, Movie S2)^20,22,23^. It is important to note that these phenotypes were often facultative and transient; a single macrophage could host an intracellular bacterial population that exhibits several distinct doubling times over the course of the acquisition (Fig. 2A–C, Figure S1). To account for this, we distinguish between the number of unique host cells (*N*_*cell*_) and the total number of measured doubling times (*N*_*dt*_). Consequently, a period of rapid intracellular growth represented a specific measured event rather than an indefinitely maintained state of unrestricted growth. Initial bacterial burden showed no correlation with subsequent bacterial doubling times within individual macrophages (Figure S2A).

Our analysis revealed that intracellular doubling time are broadly distributed, ranging from <10 h to >70 h (Fig. 2D). In untreated macrophages, Mtb WT displayed considerable heterogeneity (*CV* = 0.45, Table S1). While the distribution median of 25 h (*N*_*cells*_ = 245, *N*_*dt*_ = 402) is included in the canonical 20-25 h doubling times reported in the literature, we observed a significant subpopulation (14%) replicating rapidly ( < 16 h doubling time), alongside a dominant fraction of slow-growing events (57%). Gaussian Mixture Modelling (GMM) confirmed that the data are better described by a 2-component Gaussian mixture rather than a single distribution *(∆BIC* > 10)^36^, consisting of a distinct “actively replicating” population cantered around ~20 h and a slower population around ~45 h (Figure S3). This diversity also applied to the attenuated Mtb ∆RD1 mutant. As expected, Mtb ∆RD1 showed a reduced capacity for intracellular replication, with a median doubling time of 42 h and a sharp reduction in the total number of measurable doubling events compared to WT (*N*_*cell*_ = 83, *N*_*dt*_ = 87, Figure S1E-F). However, the fast-growing phenotype remained in 11% of the population (Fig. 2D). Fisher’s exact test (Table S2) confirmed that this proportion was statistically indistinguishable from the Mtb WT strain (*p* > 0.99), suggesting that the capability for this rapid intracellular growth may be independent of ESX-1 activity. There was also a concomitant reduction in the proportion of intercellular transfer in Mtb ∆RD1 when compared to Mtb WT (18.5% to 30.2%) as the Mtb ∆RD1 mutant does not trigger substantial cell death^30^ (Figure S1E-F, Figure S2B).

We next examined how this heterogenous growth behaviour responded to antibiotic pressure. At the population level, treatment with PZA, INH and RIF resulted in a concentration dependent reduction in measurable doubling events (Fig. 2D, Figure S1E, G). Moreover, a higher proportion of initially infected macrophages ending the acquisition uninfected (Figure S2B) and confirming the antibiotic efficacy on a bulk scale. The majority of macrophages under EC99 concentrations showed no measurable population doublings (2859 non-doubling instances and 2175 non-growing instances versus 246 macrophages with intrinsic intracellular doublings, Figure S1G). As a result, these non-growing populations were represented in the cumulative growth curves, yielding an overall flat trajectory (Figure S4A). Mtb ∆RD1 infection already resulted in a large reduction in the amount of measurable growth in untreated conditions (Figure S1F, Figure S4B) and after antibiotic treatment, the number of macrophages with intracellular doubling populations of Mtb ∆RD1 was negligible (*N*_*cell*_ = 8, across all compounds) precluding any further analysis (Figure S1H, Figure S3B, Figure S4B).

At EC50 concentration, RIF induced a marked shift toward slow growth (increasing from 57% to 86%), while PZA and INH profiles remained broadly similar to the untreated control (Fig. 2D). However, at high-dose EC99 concentration, where the vast majority of intracellular bacteria were non-doubling (Figure S1G), the rare surviving doubling populations were significantly enriched for the fast-growth phenotype. This selection was most pronounced in RIF EC99, where the proportion of fast growers changed from 14% to 64% (Fig. 2D). This resulted in an expansion of phenotypic variance (*CV* = 0.90). Pairwise Kolmogorov-Smirnov (KS) test (Figure S5) confirmed that the RIF EC99 distribution was statistically distinct from all other conditions (*D* = 0.56, *p* = <0.001 vs WT), driven by a reduction in the normal/slow growth populations and the enrichment of the fast-growing tail. In contrast, while INH EC99 and PZA EC99 treatment resulted in observable increases in the fast-growing fraction (38% and 27% respectively), their overall distributional shapes remained statistically indistinguishable from the untreated control (*p* > 0.05). However, specific analysis of the fast-growing tail revealed that these shifts were non-stochastic; pairwise Fisher’s exact tests with Benjamini-Hochberg False Discovery Rate (FDR) correction confirmed a statistically significant enrichment of the fast growing bacteria in both RIF EC99 (*p* < 0.0001) and INH EC99 (*p* < 0.01) compared to the untreated control (Table S2).

Importantly, we verified if macrophage subpopulations could explain the difference of Mtb intracellular growth. Flow-cytometry analysis was performed to correlate Mtb burden (E2-Crimson signal) and surface expression of macrophage markers (Figure S6). Our gating strategy consisted in the separation of three populations in infected samples (uninfected bystander, low or high-burden infected cells) in comparison with uninfected cells (Figure S6A). Most surface macrophage markers do not vary across the conditions, suggesting that at least by analysis of these markers, macrophage phenotypes are not associated with intracellular Mtb growth (Figure S6B-C). Taken collectively, our single-cell analysis uncovers a subpopulation of bacteria that not only replicates faster than previously reported in vitro and in cellulo but is also paradoxically enriched under specific antibiotic conditions.

### Intracellular growing Mtb populations are enriched under antibiotic pressure

**Fig. 3:**
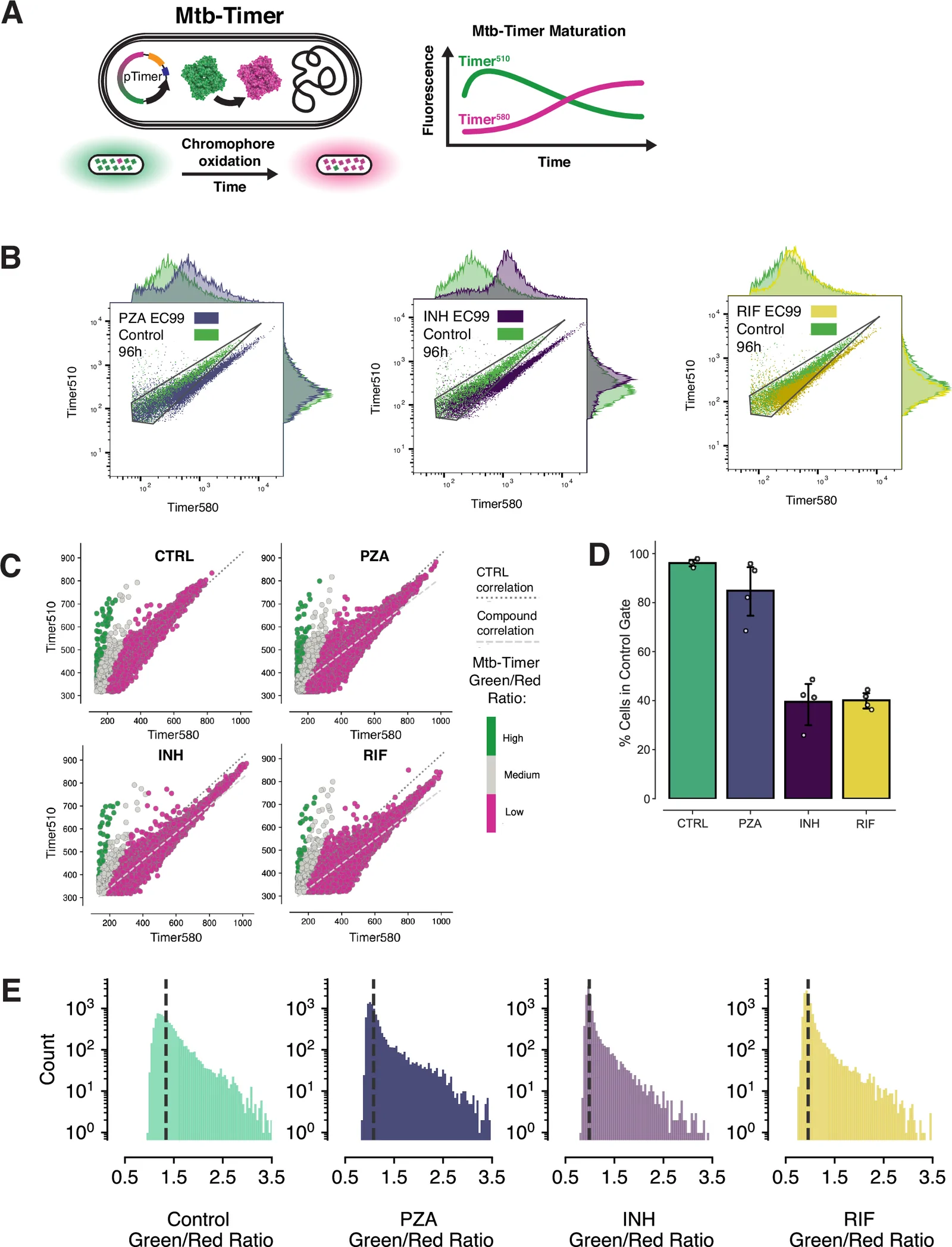
In cellulo validation of a Timer probe to measure Mtb growth. **A** Schematic of the Mtb-Timer fluorescence probe. The reporter utilises a DsRed mutant (S197T) that transitions from a green (newly synthesised) to red (mature) fluorophore over time. Rapid bacterial division dilutes the stable red fluorophore faster than the maturation rate, resulting in a high Green-to-Red (G/R) fluorescence ratio (fast growth). Conversely, slow division allows red signal accumulation, resulting in a low G/R ratio (slow growth). **B** Flow cytometry analysis of Mtb-Timer released from iPSDM at 96 h after infection. Scatter plots compare single-cell fluorescence profiles of untreated controls (green) against PZA (blue), INH (purple), and RIF (yellow) EC99 treatments. Marginal histograms display the univariate distributions for Green (Timer510) and Red (Timer580) channels. **C** Analysis of replication dynamics. Scatter plots display Green vs. Red intensity, with individual bacteria color-coded by G/R ratio category (Green: High/Fast; Grey: Medium; Magenta: Low/Slow). The dotted grey line represents the linear regression fit of the untreated control; coloured dashed lines represent the regression fit for the respective antibiotic conditions. **D** Quantification of the percentage of antibiotic-treated non-responding bacteria that fall within the “replicating” gate defined by the untreated control distribution. Data represent mean ± SD (*n* = 4 independent experiments). **E** Distribution of single-cell G/R ratios across untreated and EC99 antibiotic-treated conditions. The log-scale y-axis highlights the tail of the high G/R ratio bacteria that persist regardless of antibiotic treatment, representing a metabolically active, fast-replicating subpopulation. Vertical dashed lines indicate the median G/R ratio for each condition.

To validate the enrichment of fast-growing subpopulation after antibiotic treatment, we used an independent approach based on a Mtb H37Rv strain that expresses the fluorescent reporter Timer (Mtb-Timer)^37^. This reporter measures bacterial growth dynamics through the green-to-red (G/R) fluorescence ratio of the bacteria (Fig. 3A)^38,39^. While Mtb-Timer serves as an independent proxy of bacterial replication rate, a quantitative link between the phenomena reported in Figs. 2 and 3 remains to be established. First, to validate the functionality of the Mtb-Timer reporter in vitro, we treated Mtb with the bacteriostatic antibiotic chloramphenicol (CHL). This treatment resulted in a reduction of green intensity, demonstrating the sensitivity of the reporter to changes in Mtb replication (Figure S7B). Using flow-cytometry to analyse fluorescence intensity of Mtb in vitro, we observed a reduction in green intensity in INH and RIF, but not PZA treatment (Figure S7C), consistent with data showing that PZA requires an intracellular microenvironment to achieve full efficacy^10^. Then, using an infection workflow similar to the high-content imaging approach (Fig. 1A), we measured green and red fluorescence of in cellulo Mtb released from infected iPSDM. Upon antibiotic treatment, we observed a shift of the Mtb-Timer profile (Fig. 3B). Notably, the proportion of bacteria remaining within the control gate decreased markedly at EC99 concentrations, consistent with a bulk downshift in G/R ratio across the treated population (Figure S8B). The Mtb-Timer profile shift is more pronounced at EC99 than EC50, reflecting the concentration dependent effect of antibiotic treatment. Using the G/R ratio as a proxy for intracellular replication, we found that despite EC99 antibiotic treatment, a proportion of actively growing Mtb remained, evidenced by the persistence of high G/R ratio bacteria (green points, Fig. 3C) across all treatment conditions. The shift between the control and treatment regression lines reflects the global downshift in G/R ratio of the bulk bacterial population under antibiotic pressure. Importantly, an in vitro Mtb-Timer analysis showed minimal retention of high G/R ratio bacteria upon antibiotic treatment suggesting a host-driven component in the fast-growing phenotype of intracellular Mtb (Figure S7C). By analysing the in cellulo flow cytometry plots (Fig. 3B-C) we found that INH resulted in a shift toward higher fluorescence intensities in both green and red channels. This is consistent with INH-mediated cell wall inhibition, where bacterial division is arrested but protein translation initially continues, leading to an intracellular accumulation of reporter fluorophores due to a lack of intrabacterial dilution^32^. A similar phenotype was observed under PZA treatment (Fig. 3B-C). In contrast, RIF treatment resulted in green-red distributions similar to the control but with a specific shift in the red channel towards higher intensity (Fig. 3B-C). This agrees with the RIF mechanism of blocking mRNA synthesis, preventing the generation of new green fluorophore while the existing fluorescence reservoir continues to mature into red^32^. Across all antibiotic conditions, a consistent proportion of intracellular Mtb remained within the control gate (Fig. 3D), indicating that a subpopulation of bacteria is unlikely to be directly impacted by antibiotic treatment. Confirming the live cell imaging results, the most significant observation across all conditions was the presence of a tail of high G/R ratio bacteria, directly visualised as a persistent high G/R ratio subpopulation across all antibiotic conditions (Fig. 3E), with median G/R ratios indicated for each condition as dashed lines. Regardless of the antibiotic stress, this subpopulation confirms that a rare subset of bacteria remains metabolically active and capable of new protein synthesis. Altogether, these data show that even under EC99 antibiotic pressure, populations of growing intracellular Mtb are still present.

### Replicating Mtb subpopulations are associated with antibiotic evasion and treatment failure in vivo

**Fig. 4:**
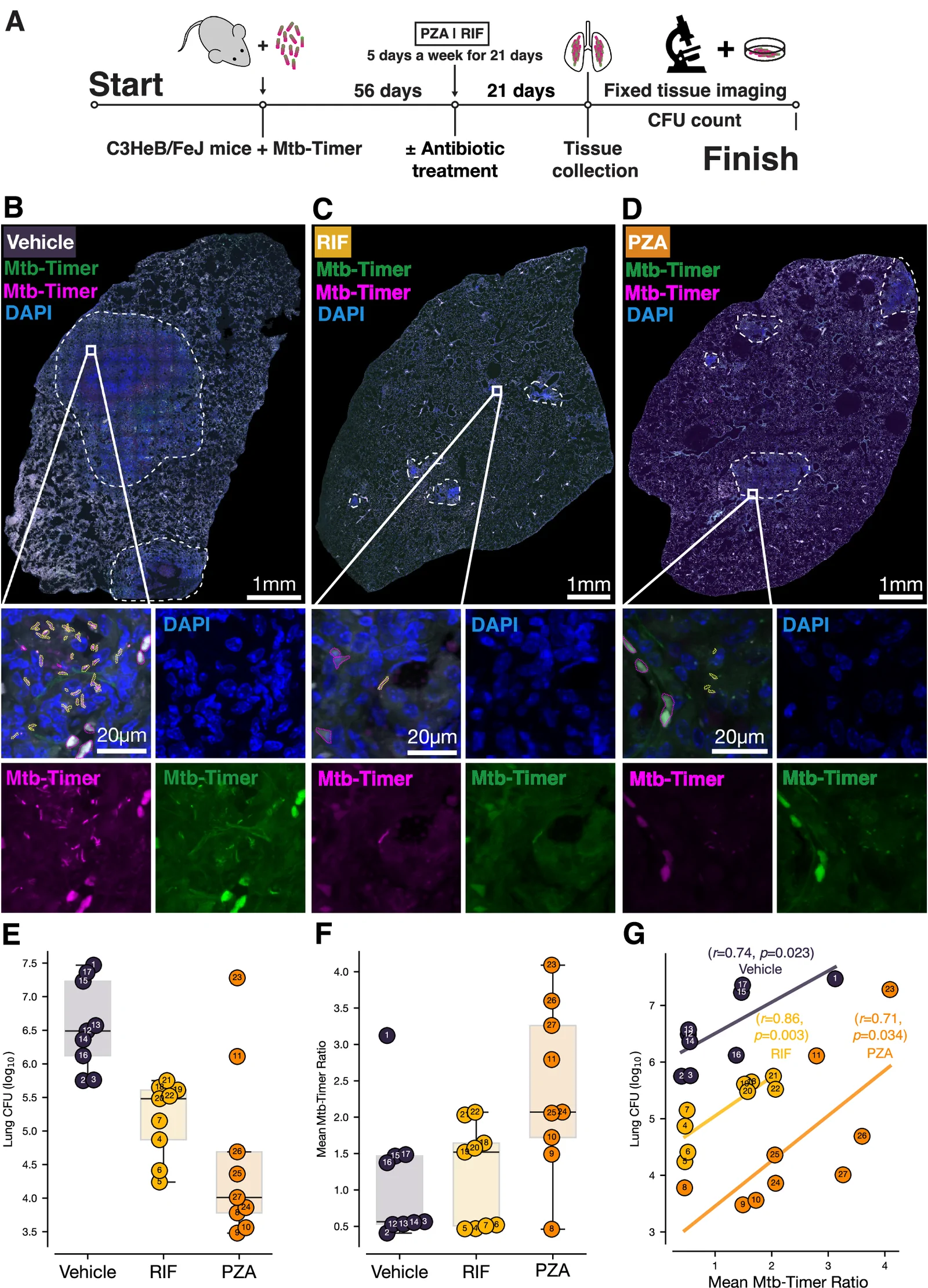
Replicating Mtb subpopulations are associated with antibiotic evasion and treatment failure in a susceptible mouse model of TB. **A** Experimental design. Susceptible C3HeB/FeJ mice were infected via aerosol with Mtb-Timer. At 56 days post-infection, mice received vehicle, Rifampicin (RIF), or Pyrazinamide (PZA) for 3 weeks prior to lung harvest for CFU enumeration and high-resolution imaging. **B–D** Representative confocal microscopy of lung lobectomies from Vehicle (B), RIF (C), and PZA (D) treated mice. Large tissue scans display gross pathology and bacterial distribution (DAPI: blue; Mtb-Timer: Green/Red; Lesions: white dashed line). Insets reveal single-cell heterogeneity of intracellular bacteria fluorescence profile, with individual bacterial cells indicated by yellow dashed lines. Structures manually excluded from quantification, including erythrocytes and tissue artefacts, are indicated by magenta dashed lines. **E** Quantification of total bacterial burden via lung CFU counts (log_10_), showing reduction upon antibiotic treatment. **F** Quantification of single-cell replication dynamics (Mtb-Timer Green/Red ratio) across treatment groups. While RIF and Vehicle show relatively tight distributions, PZA treatment reveals marked heterogeneity, with a subset of mice retaining bacterial populations with very high replication (high G/R ratio). **G** Correlation analysis between bacterial burden (Lung CFU) and replication (Mean Mtb-Timer Ratio). Scatter plot reveals a significant positive correlation across all conditions: vehicle (*r* = 0.74, *p* = 0.023), RIF (*r* = 0.86, *p* = 0.003) and PZA (*r* = 0.71, *p* = 0.034). The solid lines represent the linear regression of each group. Two-way ANOVA was used to assess batch variance across experiments, supporting a biological origin for the observed inter-individual heterogeneity prior to data pooling. Data points represent independent biological replicates (individual mice; *n* = 9 per group). Data are pooled from two independent experiments (*n* = 4 and *n* = 5 mice per group, respectively. Numbers within data points denote individual mouse identifiers to track biological variance across panels. For all box plots, the centre line represents the median, box bounds represent the 25th and 75th percentiles, and whiskers extend to the minimum and maximum values within 1.5 times the interquartile range.

To confirm the fast growth phenotype, we next sought to validate these dynamics in vivo with Mtb-Timer. Based on our observations in cellulo, we hypothesised that the presence of fast-growing intracellular Mtb populations is responsible for poor antibiotic response in vivo. To investigate this, we infected C3HeB/FeJ mice, a susceptible model of TB disease^40,41^, with the reporter strain Mtb-Timer. After 56 days of infection, we treated the mice with vehicle, RIF or PZA for 3 weeks before collecting the lungs for Colony Forming Units (CFU) counting and high-resolution confocal imaging (Fig. 4A). To quantify the replication dynamics (G/R ratio) at the single-cell level in the lungs, we developed a semi-automated pipeline that integrates manual bacterial ROI annotation with automated host-cell segmentation in QuPath^42^ (Figure S9). Consistent with standard efficacy, both RIF (*p* < 0.01) and PZA (*p* < 0.001) treatment significantly reduced the total bacterial burden compared to vehicle controls as measured by bulk CFU counts (Fig. 4E, Table S3). As previously reported, PZA efficacy was highly heterogeneous^43^, with a subset of exhibiting poor control of infection as measured by CFU (Fig. 4E-F).

Strikingly, our single-cell analysis revealed that the bacterial populations surviving antibiotic treatment sustained replication that were either comparable to (RIF, *p* = 0.97) or significantly higher than (PZA, *p* < 0.05) those in the vehicle-treated controls (Fig. 4F, Table S4). This uncoupling of burden and replication was confirmed by a positive correlation between the G/R ratio and the lung CFU across all conditions (Vehicle: *r* = 0.74, *p* = 0.023; RIF: *r* = 0.86, *p* = 0.003; PZA: *r* = 0.71, *p* = 0.034; Fig. 4G). Notably, in the antibiotic treated groups, the specific mice that failed to respond to therapy (particularly in PZA conditions) displayed the highest proportion of fast-replicating bacteria (Fig. 4G). Altogether, these findings show that growing intracellular Mtb populations are associated with antibiotic treatment failure in vivo.

## Discussion

Here, we reveal that Mtb has the facultative capacity to adopt a remarkably wide range of intracellular growth phenotypes that are relevant for antibiotic treatment. Whilst other approaches have previously described such heterogeneity in vitro, in cellulo and in vivo^12,22,23,26,44,45^, our approach is capable of tracking individual infected macrophage populations and quantifying intracellular Mtb growth rates in the relevant human macrophage host whilst also under antibiotic pressure. Macrophages harbouring fast-growing Mtb are not merely theoretical outliers but remain during the infection, as Mtb permissive macrophages have been reported in human TB^7^ and mouse models of TB^15,44^. Our results agree with the observed intracellular growth of Mtb in animal models where the logarithmic increase of bacteria does not always match a slow growth rate^40^.

Antibiotic phenotypic tolerance is conventionally associated with the slow (or non) growth of Mtb and other bacteria under host environmental stress^46^. Unexpectedly, we show that a distinct subpopulation of fast-growing Mtb survives and is specifically enriched under antibiotic pressure, likely without developing resistance mutations and mostly owing to phenotypic changes triggered by the intracellular milieu^47,48^. Our data agrees with reports in *E. coli*, where fast-growing resilience has been postulated as a mechanism of antibiotic escape in vitro^49,50^. It is possible that either macrophages or rapidly replicating Mtb fail to internalise effective concentrations of antibiotics^49^. Alternatively, infection may enhance the efflux capacity of the host macrophages or intracellular Mtb, subsequently reducing antibiotic accumulation^51^.

Several factors lead to RIF tolerance, heteroresistance, or persistence^52^. Our observation that RIF EC99 treatment expands the phenotypic variance (*CV* = 0.90) suggests a “bet-hedging” strategy, where the bacteria access a high-risk, high-reward state of rapid replication to survive. Our data raise the possibility that fast-replicating Mtb populations might evade RIF by reducing the accessibility or binding efficacy of RIF to RNA polymerase due to heightened transcriptional activity. Moreover, certain subpopulations of Mtb can continue to grow and divide in the presence of RIF due to RIF-induced upregulation of the *rpoB* gene^53^. Our findings in Mtb align with mathematical models predicting that bactericidal antibiotics targeting fast-growing bacteria leave a small but largely viable bacterial population^54^.

INH inhibits mycolic acid synthesis and predominantly acts on replicating bacteria. The evasion of rapidly dividing populations suggests that accelerated mycolic acid production may exceed the inhibitory capacity of INH, consistent with previous observations of growth rate-dependent antibiotic evasion^55,56^. This could be related to the postulated bacterial shape dependent resistance to antibiotics^57^. The efficacy of INH is also impacted by different host environmental factors, particularly pH and redox^58^. Different from our data with Mtb in macrophages, in in vitro *M. smegmatis*, there is no correlation between single-cell growth rates and cell fates as both slowly and rapidly growing bacteria likely persisted after INH treatment^59^. These differences could be due to the stress imposed by the host cell^46^.

PZA has been proposed to disrupt multiple bacterial pathways and is known for being effective against both replicating and non-replicating populations after its bioconversion into pyrazinoic acid (POA)^60^. Increased metabolic activity in rapidly growing populations of Mtb could enhance efflux pump activity and thus expel the POA prior to any bactericidal effects. Alternatively, an increase in cell volume could contribute to the dilution of drug concentration^49^. Contrary to RIF and INH, we did not observe an enrichment in fast-growing Mtb in our in cellulo analysis. However, our in vivo findings, where PZA treatment failed to eliminate bacteria with high G/R ratios in susceptible mice, indicating resilience is a major barrier to clearance in the lung. These observations suggest that in vivo environmental conditions play an important role in PZA activity. We speculate that the fast-growing Mtb subpopulation, which represents a minor fraction of the total intracellular population in cellulo (Fig. 2D, Figure S1G), may escape antibiotic clearance under prolonged treatment pressure in vivo. In the context of treatment failure, this subpopulation would become increasingly predominant, consistent with the elevated G/R ratios observed in high-burden mice (Fig. 4F, G). Given that PZA possibly has many mechanisms of action^31,61,62^, future studies are necessary to define the relationship between Mtb growth and PZA efficacy. Our studies uncover an additional phenomenon of antibiotic evasion in a subpopulation of fast-growing intracellular Mtb, alongside non-growing or slow-growing populations. Our findings have potential implications for TB chemotherapy. These observations raise the possibility that some anti-TB antibiotics may fail to target fast-growing populations effectively or that these fast-growing populations evade antibiotic treatment. Further understanding of the biology and resilience of these fast-growing intracellular populations is crucial for designing improved TB treatment strategies.

## Methods

### *M. tuberculosis* culture

Fluorescent *M. tuberculosis* H37Rv pTEC19 WT and ΔRD1 strains were used in this study^10^. *M. tuberculosis* H37Rv pGMEH-Pml-DsRed1E5-0 × 0 WT strain (Mtb-Timer) was generated in this study by electroporation as previously described^63^. All strains were grown in Middlebrook 7H9 broth supplemented with 0.2% glycerol, 0.05% Tween-80, 10% ADC and 50 mg/L Hygromycin B as a selection marker for fluorescent strains. Bacterial cultures were incubated at 37 °C with rotation in 50 mL conical tubes.

### Human iPSC culture

KOLF2-C1 AAVS1:EGFP human iPSC were a gift from the Skarnes Lab (The Jackson Laboratory, CT, USA) and were generated as previously described^28^. Briefly, the KOLF2-C1 AAVS1 cells express a EGFP reporter under the control of the strong CAGG promoter in the safe harbour locus AAVS1. The cells were maintained in Vitronectin XF (StemCell Technologies, 100-0763)-coated plates with Essential 8 medium (Gibco, A1517001). Cells were authenticated by STR profiling and checked regularly for *Mycoplasma* contamination. Cells were passaged using Versene (Gibco, 15040066).

### iPSDM differentiation

Monocyte factories were set up following a previously reported protocol^29^. Embryoid bodies (EBs) were fed daily with two times 50% medium changes with E8 supplemented with 50 ng mL^−1^ hBMP4 (Peprotech, 120-05), 50 ng ml^−1^ hVEGF (Peprotech, 100-20) and 20 ng mL^−1^ hSCF (Peprotech, 300-07) for 3 days. On day 4, the EBs were collected and seeded at 100-150 EBs per T175 or 250-300 per T225 flask in XVIVO-factory medium. These monocyte factories were fed weekly for 5 weeks until monocytes were observed in the supernatant. Up to 50% of the supernatant was collected weekly and centrifuged at 300 *g* for 5 min. The cells were resuspended in X-VIVO-differentiation medium. Monocytes were plated at 5 × 10^6^ cells per 10 cm Petri dish to differentiate over 7 days; on day 4, a 50% medium change was performed. To detach, cells were washed once with PBS (pH 7.4), then incubated with Versene for 15 min at 37 °C and 5% CO_2_ before diluting 1:3 with PBS and gently scraping. Macrophages were centrifuged at 300 *g* and plated for experiments.

### Infection of iPSDM with *M. tuberculosis*

For the high-content, live-cell imaging, induced pluripotent stem cell derived macrophages (iPSDM) were seeded at a density of 50,000 cells per well of an olefin-bottomed 96-well plate (Phenoplate, Revvity 605532). For flow cytometry, iPSDM were seeded at density of 500,000 cells per well on a tissue culture-treated 12-well plate (ThermoFisher, 3513). Mid-logarithmic-phase bacterial cultures (OD_600_ 0.5–1.0) were centrifuged at 2,000 *g* for 5 min and washed twice in PBS. Pellets were then shaken vigorously for 1 min with 2.5–3.5 mm glass beads (VWR, 332124 G) and bacteria resuspended in 10 mL macrophage culture medium before being centrifuged at 300 *g* for 5 min to remove large clumps. The top 7 mL of bacterial suspension was taken, the OD_600_ was measured, and the suspension was diluted appropriately for infection. After 2 h of uptake, extracellular bacteria were removed with two washes in PBS and macrophages were incubated at 37 °C and 5% CO_2_ for 24 h. Then, media was changed with media containing or not antibiotics before proceeding to the subsequent live-cell imaging.

### In cellulo antibiotic treatments

Antibiotic concentrations used in this study, defined as in cellulo EC50 and EC99, were previously determined^10,64^. The values of EC50 for PZA (Sigma, PHR1576-500mg), RIF (Sigma, R3501-1g) and INH (Sigma, I3377-5G) were respectively 60 μg/mL, 0.1 μg/mL and 0.04 μg/mL. The values of EC99 for PZA, RIF and INH were respectively 400 μg/mL, 2 μg/mL and 2 μg/mL. Antibiotics were added into the infected macrophages after 24 h of infection. For in vitro antibiotic treatment of Mtb-Timer, late exponential phase Mtb culture were diluted to OD_600_ = 0.1 and incubated at 37 °C with rotation for 2 days. Cultures were then supplemented with PZA, INH, RIF (at EC99) and CHL^65^ (10 µg/mL) and incubated for an additional 3 days at 37 °C with rotation.

### In cellulo image acquisition

Image acquisition was achieved with the OPERA Phenix high-content, confocal, incubator microscope with a 40x water-immersion 1.1NA objective. A single image of 323 µm × 323 µm was acquired across 9 contiguous positions which were compiled together into a 3 × 3 mosaic of 904 µm × 904 µm with a 10% overlap between tiles. This tessellated field of view was repeated across 3 × 2 µm z-slices and the 2× green/red imaging channels. The channel acquisition parameters were **λ**_ex_ = 488, **λ**_em_ = 522 (exposure time 100 ms) for KOLF-EGFP macrophages and **λ**_ex_ = 640, **λ**_em_ = 706 (exposure time 200 ms) for E2-Crimson-Mtb. This was then repeated every 30/60 min for 150/75 frames to reach a total timelapse acquisition of 75 h. This process was repeated over 42 different imaging wells, each with a different Mtb strain or antibiotic condition within, resulting in a final acquisition of 340,200 images (2.58TB) per biological repeat. A total of 4 biological repeats were conducted. The untiled images were then exported out of the Harmony software (Revvity, version 4.9) to allow for a an open-source Python-based image analysis (v3.10.19).

### In cellulo image analysis

The first step in the Python-based image analysis was to tile the images into full field-of-view mosaics. This facilitated the tracking of macrophages across individual tiles, greatly increasing the number of tracked macrophages in our final dataset. This tiling was achieved using a bespoke Dask (v2026.1.2) Python script, based on the DaskFusion repository^66^, that extracts the Cartesian coordinates of each image tile from the OPERA Phenix metadata and stiches them together into a Dask image volume^67^. This lazy-loading approach enabled the selective rendering of individual frames, z-slices, or cropped regions of interest, conserving computational power by avoiding the need to load the entire 62.95GB-per-condition image volume for each visual examination or partial analysis.

The next step in the analysis was to segment the cytoplasmic extent of the macrophages. Due to the inherent heterogeneity of macrophage morphology, we decided to train our own segmentation model using Cellpose 2 (v2.3.2)^68^. We manually segmented 4934 individual cell segments across a range of 72 different images, representing a diverse range of time points, cellular densities, infection statuses and drug conditions. The resulting self-trained model improved the Jaccard index (a measure of the number of objects correctly identified) by 25% and the intersection over union (IoU, a measure of the accuracy of individual segments) by 2.2%. The increase in the Jaccard index metric highlights an improvement upon the primary limitation of using the pre-trained model, which would frequently over- or under-segment cells into merged- or multiply- fragmented instances. The marginal improvement of the IoU indicates that the boundary accuracy of individual segments was already robust, underscoring the quality of the Cellpose generalist approach.

After segmentation, each cell was localised and tracked using the btrack multi-object tracking algorithm (v0.7.0)^69^. This localisation step included the quantification of several key cellular properties, most importantly the total intracellular Mtb content. This was defined as the area of E2-Crimson fluorescence above a threshold pixel value (480) that was obtained through manual segmentation of Mtb using a custom napari^35^ (v.0.5.6) key-binding script. These cellular properties were then stored, along with the centroid and time coordinates of each segment, as Pytracklet objects that serve as the input to the tracking. The btrack algorithm utilises a combination of a motion model and a visual feature comparison to predict the movement of each cell and compare the visual properties measured in the localisation step across frames to ensure that each segment is accurately linked to its correct temporal evolution. The result of this is a time series of united objects that chart the trajectory of a single macrophage with associated properties, including intracellular Mtb burden.

The final step in the image analysis pipeline was to process the time-series information of intracellular Mtb content for each cell into growth curves, from which single-cell intracellular doubling amounts and times could be extracted. This firstly involves a linear interpolation of the data to account for points at which the segmentation may have been missed, or image frame skipped. A nonparametric locally weighted scatterplot smoothing (LOWESS) model (statsmodels v0.14.6)^70^ was then applied to this time series to generate a smooth, continuous curve. This allowed for a reliable estimation of intracellular Mtb doubling times as it removes any fluctuations from the data that could result in an inaccurate assessment of population doubling. The coefficient of determination (R^2^) between the raw intracellular Mtb signal and LOWESS model was used to isolate potential instances of extrinsic Mtb growth, with trajectories showing R^2^ < 0.7 being subsequently validated as either extracellular transfer or uptake using a napari-based human-in-the-loop inspection of single-cell instances. The threshold of 0.7 was empirically determined by inspecting several hundred growth patterns and their associated R^2^ scores.

### Flow cytometry and data analysis

For in cellulo Mtb-Timer experiments, infected iPSDM at 2 h, 24 h and 96 h post-infection were washed once with PBS and lysed in 0.05% Triton-X100 in H_2_O at 37 °C for 5 min. Released Mtb was fixed in 4% PFA in PBS at 4 °C overnight. For in vitro Mtb-Timer experiments, at the experimental endpoint of antibiotic treatment, Mtb cultures were pelleted by centrifugation and fixed in 4% PFA in PBS at 4 °C overnight. Fixed in vitro and in cellulo Mtb were collected by centrifugation, washed in PBS, and resuspended in PBS + 1% BSA.

For cell surface quantification of macrophage markers, iPSDM were detached and resuspended in 1% BSA in PBS and stained with fluorescently conjugated antibodies against CD11B (1:20, Thermo Fisher Scientific, 14-0112-82), CD14 (1:20, BD Biosciences, 555399), CD86 (1:20, BD Biosciences, 562433), CD119 (1:20, BD Biosciences, 558934), CD163 (1:20, BD Biosciences, 563697), CD169 (1:20, BD Biosciences, 565248) and CD206 (1:20, BD Biosciences, 566281) on ice for 1 hour. The stained cells were collected by centrifugation, washed in PBS, and resuspended in PBS + 1% BSA.

Samples were acquired on a BD LSRFortessa Cell Analyzer (BD Biosciences) using BD FACSDiva software (v8.0.1) and analysed using FlowJo (FlowJo, LLC, v10.10.0). At least 10,000 events per condition were recorded.

### Murine aerosol *M. tuberculosis* infection and antibiotic treatment

*M. tuberculosis* H37Rv wild type (WT) expressing Timer was used to infect mice. Bacteria were verified by sequencing and tested for phthiocerol dimycocerosates (PDIM) positivity using thin-layer chromatography. C3HeB/FeJ mice (*Mus musculus*) were bred under specific pathogen-free conditions at The Francis Crick Institute (London, UK). Female mice were housed in cages within isolators in the BSL-3 facility under controlled conditions of temperature (19–23 °C) and humidity (55 ± 10%) on a 12 h light/12 h dark cycle, with food and water provided *ad libitum*. Animal studies and breeding were approved by the Francis Crick Institute Animal Welfare and Ethical Review Body (AWERB) and performed under UK Home Office project licence P4D8F6075, in accordance with the Animals (Scientific Procedures) Act 1986. Infections were performed across two independent batches (batch 1: 3 vehicle, 4 RIF, 4 PZA; batch 2: 6 vehicle, 5 RIF, 5 PZA; *n* = 9 per condition) in the category 3 animal facility of the Francis Crick Institute. For aerosol infection, Mtb-Timer was grown to mid-log phase (OD600 = 0.6) in 7H9 supplemented with ADC, Tween 20 and 50 µg/mL Hygromycin. An infection sample was prepared from this to enable delivery of approximately 100 CFU per mouse lung using a modified Glas-Col aerosol infection system. Treatment of mice commenced at day 56 post-infection, by administration of either antibiotic or diluent control, performed daily for 5 days a week via oral gavage over 3 weeks. The antibiotic treatment groups were given either 30 mg/kg PZA (in water) or 10 mg/kg rifampicin (in 1% DMSO); control groups received water or 1% DMSO. At day 77 post-infection, mice were culled and the lungs were obtained. The left lobe from each mouse lung was excised and perfused with 10% neutral-buffered PFA. The remaining lung lobes were used to determine bacterial counts by plating serial dilutions of homogenates on duplicate 7H11 agar plates, supplemented with OADC and containing 50 µg/mL Hygromycin. Colonies were counted 2 to 3 weeks after incubation at 37 °C.

### Lung sectioning and imaging

Preparation of cryosections: Lungs were equilibrated in 0.1 M HEPES buffer (pH 7.4) with 0.2 M sucrose overnight before being transferred to silicon molds containing OCT medium (Agar scientific, AGR1180). Molds containing OCT and lungs were then transferred to dry ice and frozen in preparation for sectioning. Sections were cut using a Leica CM30505S Cryostat (CT-18 °C, OT-20 °C) to a size of 8 μm. These sections were collected on SuperFrost Plus Adhesion slides (Thermo Fisher, 11950657) and stored at −80 °C before further processing. Tissue sections on SuperFrost Plus Adhesion slides (Thermo Fisher Scientific, 11950657) were thawed for approximately 20 min on a heat block set to 50 °C. A hydrophobic border was drawn around the tissue sections to prevent solution overflow. The slides were then washed with PBS and quenched with 50 mM NH_4_Cl for 10 min to reduce autofluorescence. Following another wash with PBS, DAPI (1:10,000) in PBS was added for 20 min to stain the nuclei. After a final wash with PBS, the slides were allowed to air-dry slightly before mounting with Dako Fluorescence Mounting Medium (Agilent, S3023). Images were acquired at 40x magnification using a Olympus CSU-W1 SoRa Spinning Disk Microscope (Olympus). Scans were saved as.ets file and visualised using Qupath^42^.

### Single cell tissue image analysis

Images were loaded on QuPath^42^ (v0.51) and cells were segmented using the “cell detection” module on the DAPI channel. This approach detected the nuclei and then allowed for a perinuclear expansion of 5 μm to determine each cell boundary. Bacteria were segmented with the QuPath brush annotation in a semi-automated manner, using GFP and DsRed channel as the input. The cytoplasmic and Mtb segmentation masks were then exported to OME-NGFF Zarr^71^ for batch quantification of single-cell Mtb levels using Python.

### Statistical analysis

Statistical analyses were performed using Python scipy.stats (v1.15.2) and scikit-learn (v1.7.2).). To quantify the spread and heterogeneity of replication dynamics, we calculated the Coefficient of Variation (*CV* = *σ/µ*) for all doubling time distributions. Changes in *CV* were used to assess the contraction or expansion of phenotypic heterogeneity under antibiotics. To specifically compare the shapes of doubling times distributions and identify distinct phenotypic clusters, we performed pairwise Kolmogorov-Smirnov (KS) tests. The resulting KS statistics (*D*) were visualized as a matrix to reveal the degree of similarity between conditions (Figure S5). To deconstruct the underlying subpopulations of growth phenotypes within the heterogeneous doubling time data, we applied Gaussian Mixture Models (GMM) using the Expectation-Maximisation (EM) algorithm (sklearn.mixture.GaussianMixture). The optimal number of components (*k*) was determined by minimising the Bayesian Information Criterion (BIC). To statistically compare the proportions of categorical growth phenotypes (Fast, Intermediate, Slow) between conditions, we utilised Fisher’s Exact Test. This method was selected over Chi-Squared to provide exact *p*-values for specific pairwise comparisons of rare subpopulations (e.g., fast growers in RIF-treated vs. Control). *P*-values for multiple comparisons were adjusted using the Benjamini-Hochberg False Discovery Rate (FDR) method where appropriate. For in vivo single-cell analysis, the relationship between bacterial burden (Red fluorescence) and replication rate (Green/Red ratio) was assessed using Pearson’s correlation coefficient (scipy.stats.pearsonr).

### Visualisation

Visualisation of timelapse imagery, accompanying segmentation and tracked macrophage populations were all conducted within napari^35^ utilising OME-NGFF Zarr^71^ and Dask^72^ for efficient lazy-loading of large image arrays. Cropped video glimpses of macrophages and Mtb were compiled as RGB.mp4 video files to facilitate the iterative loading and classification of macrophage phenotypes using napari key-bindings. Flow cytometry plots were generated using FlowJo and the Seaborn (v0.13.2)^73^ package in Python. In vivo data was visualised using napari^35^ and QuPath^42^.

### Reporting summary

Further information on research design is available in the Nature Portfolio Reporting Summary linked to this article.

## Supplementary information


Suppl information
Description of Additional Supplementary Files
Movie S1
Movie S2
Movie S3
Reporting Summary
Transparent Peer Review file


## Source data


Source Data


## Data Availability

The processed single-cell trajectory data generated in this study (singlecell_trajectories_dataframe.parquet), comprising >20,000 single-cell trajectories across 121 fields of view and 4 biological replicates, have been deposited in Zenodo under DOI 10.5281/zenodo.20831464 (https://doi.org/10.5281/zenodo.20831464)^74^. A small, downscaled example acquisition sufficient to run the analysis pipeline end to end is included in the macrohet code repository (https://github.com/nthndy/macrohet)^75^. The full raw imaging data ( ~ 35 TB) are not held in a public repository owing to their size and are available under restricted access from the corresponding author. Requests will be responded to within six weeks, with data shared by arranged institutional transfer, and the data will be retained for at least 10 years following publication. There are no restrictions on who may request the data or the purpose for which they may be used. Source data for all main-text and Supplementary Figs. are provided with this paper. Source data are provided with this paper.

## Code availability

The analysis code used to generate the dataset and to reproduce all live-cell analyses is available in the macrohet repository (https://github.com/nthndy/macrohet) and archived in Zenodo under DOI 10.5281/zenodo.20819398 (https://doi.org/10.5281/zenodo.20819398)^75^. The repository includes a small, downscaled example acquisition that allows the segmentation, tracking and quantification pipeline to be run end to end without the full imaging dataset.

## References

[1] Daniel, T. M. The history of tuberculosis. *Respir. Med.* **100**, 1862–1870 (2006). 16949809 10.1016/j.rmed.2006.08.006

[2] WHO. Global Tuberculosis Report 2025. *Geneva, World Health Organization* (2025).

[3] Dorman, S. E. et al. Four-Month Rifapentine Regimens with or without Moxifloxacin for Tuberculosis. *N. Engl. J. Med.* **384**, 1705–1718 (2021). 33951360 10.1056/NEJMoa2033400 PMC8282329

[4] Fox, W., Ellard, G. A. & Mitchison, D. A. Studies on the treatment of tuberculosis undertaken by the British Medical Research Council tuberculosis units, 1946-1986, with relevant subsequent publications. *Int J. Tuberc. Lung Dis.* **3**, S231–S279 (1999). 10529902

[5] Dartois, V. A. & Rubin, E. J. Anti-tuberculosis treatment strategies and drug development: challenges and priorities. *Nat. Rev. Microbiol.* **20**, 685–701 (2022). 35478222 10.1038/s41579-022-00731-y PMC9045034

[6] Connolly, L. E., Edelstein, P. H. & Ramakrishnan, L. Why is long-term therapy required to cure tuberculosis? *PLoS Med.* **4**, e120 (2007). 17388672 10.1371/journal.pmed.0040120 PMC1831743

[7] Kaplan, G. et al. Mycobacterium tuberculosis growth at the cavity surface: a microenvironment with failed immunity. *Infect. Immun.* **71**, 7099–7108 (2003). 14638800 10.1128/IAI.71.12.7099-7108.2003 PMC308931

[8] Chung, E. S., Johnson, W. C. & Aldridge, B. B. Types and functions of heterogeneity in mycobacteria. *Nat. Rev. Microbiol* **20**, 529–541 (2022). 35365812 10.1038/s41579-022-00721-0 PMC9681535

[9] Dhar, N., McKinney, J. & Manina, G. Phenotypic Heterogeneity in Mycobacterium tuberculosis. *Microbiol. Spectr.* **4** 10.1128/microbiolspec.TBTB2-0021-2016 (2016). 27837741

[10] Santucci, P. et al. Intracellular localisation of Mycobacterium tuberculosis affects efficacy of the antibiotic pyrazinamide. *Nat. Commun.* **12**, 3816 (2021). 34155215 10.1038/s41467-021-24127-3 PMC8217510

[11] Greenwood, D. J. et al. Subcellular antibiotic visualization reveals a dynamic drug reservoir in infected macrophages. *Science* **364**, 1279–1282 (2019). 31249058 10.1126/science.aat9689 PMC7012645

[12] Aljayyoussi, G. et al. Pharmacokinetic-Pharmacodynamic modelling of intracellular Mycobacterium tuberculosis growth and kill rates is predictive of clinical treatment duration. *Sci. Rep.* **7**, 502 (2017). 28356552 10.1038/s41598-017-00529-6 PMC5428680

[13] Tulkens, P. M. Intracellular distribution and activity of antibiotics. *Eur. J. Clin. Microbiol Infect. Dis.* **10**, 100–106 (1991). 1864271 10.1007/BF01964420

[14] Barcia-Macay, M., Seral, C., Mingeot-Leclercq, M. P., Tulkens, P. M. & Van Bambeke, F. Pharmacodynamic evaluation of the intracellular activities of antibiotics against Staphylococcus aureus in a model of THP-1 macrophages. *Antimicrob. Agents Chemother.* **50**, 841–851 (2006). 16495241 10.1128/AAC.50.3.841-851.2006 PMC1426441

[15] Gill, W. P. et al. A replication clock for Mycobacterium tuberculosis. *Nat. Med.* **15**, 211–214 (2009). 19182798 10.1038/nm.1915 PMC2779834

[16] Sarathy, J. P. et al. Extreme Drug Tolerance of Mycobacterium tuberculosis in Caseum. *Antimicrob. Agents Chemother.* **62**, 10.1128/AAC.02266-17 (2018). PMC5786764 29203492

[17] Keren, I., Minami, S., Rubin, E. & Lewis, K. Characterization and transcriptome analysis of Mycobacterium tuberculosis persisters. *mBio* **2**, e00100–e00111 (2011). 21673191 10.1128/mBio.00100-11 PMC3119538

[18] Herbert, D. et al. Bactericidal action of ofloxacin, sulbactam-ampicillin, rifampin, and isoniazid on logarithmic- and stationary-phase cultures of Mycobacterium tuberculosis. *Antimicrob. Agents Chemother.* **40**, 2296–2299 (1996). 8891133 10.1128/aac.40.10.2296 PMC163523

[19] Tailleux, L. et al. Constrained intracellular survival of Mycobacterium tuberculosis in human dendritic cells. *J. Immunol.* **170**, 1939–1948 (2003). 12574362 10.4049/jimmunol.170.4.1939

[20] Mahamed, D. et al. Intracellular growth of Mycobacterium tuberculosis after macrophage cell death leads to serial killing of host cells. *Elife* **6**, 10.7554/eLife.22028 (2017). PMC5319838 28130921

[21] Repasy, T. et al. Intracellular bacillary burden reflects a burst size for Mycobacterium tuberculosis in vivo. *PLoS Pathog.* **9**, e1003190 (2013). 23436998 10.1371/journal.ppat.1003190 PMC3578792

[22] Chung, E. S., Kar, P., Kamkaew, M., Amir, A. & Aldridge, B. B. Single-cell imaging of the Mycobacterium tuberculosis cell cycle reveals linear and heterogenous growth. *Nat. Microbiol.* 10.1038/s41564-024-01846-z (2024). 39548343 PMC11602732

[23] Toniolo, C., Sage, D., McKinney, J. D. & Dhar, N. Quantification of Mycobacterium tuberculosis Growth in Cell-Based Infection Assays by Time-Lapse Fluorescence Microscopy. *Methods Mol. Biol.* **2813**, 167–188 (2024). 38888778 10.1007/978-1-0716-3890-3_12

[24] Dartois, V. & Barry, C. E. 3rd A medicinal chemists’ guide to the unique difficulties of lead optimization for tuberculosis. *Bioorg. Med. Chem. Lett.* **23**, 4741–4750 (2013). 23910985 10.1016/j.bmcl.2013.07.006 PMC3789655

[25] Day, N. J., Santucci, P. & Gutierrez, M. G. Host cell environments and antibiotic efficacy in tuberculosis. *Trends Microbiol* **32**, 270–279 (2024). 37709598 10.1016/j.tim.2023.08.009

[26] Mistretta, M. et al. Dynamic microfluidic single-cell screening identifies pheno-tuning compounds to potentiate tuberculosis therapy. *Nat. Commun.* **15**, 4175 (2024). 38755132 10.1038/s41467-024-48269-2 PMC11099131

[27] Jovanovic, A. et al. Large-scale testing of antimicrobial lethality at single-cell resolution predicts mycobacterial infection outcomes. *Nat. Microbiol*. 10.1038/s41564-025-02217-y (2026). 41513995 PMC12872448

[28] Skarnes, W. C., Pellegrino, E. & McDonough, J. A. Improving homology-directed repair efficiency in human stem cells. *Methods* **164-165**, 18–28 (2019). 31216442 10.1016/j.ymeth.2019.06.016

[29] Bernard, E. M. et al. M. tuberculosis infection of human iPSC-derived macrophages reveals complex membrane dynamics during xenophagy evasion. *J. Cell Sci.* **134** 10.1242/jcs.252973 (2020). PMC7710011 32938685

[30] Lerner, T. R. et al. Mycobacterium tuberculosis replicates within necrotic human macrophages. *J. Cell Biol.* **216**, 583–594 (2017). 28242744 10.1083/jcb.201603040 PMC5350509

[31] Zhang, Y., Wade, M. M., Scorpio, A., Zhang, H. & Sun, Z. Mode of action of pyrazinamide: disruption of Mycobacterium tuberculosis membrane transport and energetics by pyrazinoic acid. *J. Antimicrob. Chemother.* **52**, 790–795 (2003). 14563891 10.1093/jac/dkg446

[32] Somoskovi, A., Parsons, L. M. & Salfinger, M. The molecular basis of resistance to isoniazid, rifampin, and pyrazinamide in Mycobacterium tuberculosis. *Respir. Res.* **2**, 164–168 (2001). 11686881 10.1186/rr54 PMC2002067

[33] Palomino, J. C. & Martin, A. Drug Resistance Mechanisms in Mycobacterium tuberculosis. *Antibiotics (Basel)* **3**, 317–340 (2014). 27025748 10.3390/antibiotics3030317 PMC4790366

[34] Cleveland, W. S. Robust Locally Weighted Regression and Smoothing Scatterplots. *J. Am. Stat. Assoc.* **74**, 829–836 (1979).

[35] Sofroniew, N. L. et al. *napari: a multi-dimensional image viewer for Python*, https://github.com/napari/napari (2019).

[36] Kass, R. E. & Raftery, A. E. Bayes Factors. *J. Am. Stat. Assoc.* **90**, 773–795 (1996).

[37] Campo-Perez, V., Luk, C. H., Botella, L., Vaubourgeix, J. & Gutierrez, M. G. Mtb-Timer: a fluorescent reporter to visualize Mycobacterium tuberculosis replication and antibiotic responses. *mSystems*, e0179625, 10.1128/msystems.01796-25 (2026). PMC13288934 42153669

[38] Claudi, B. et al. Phenotypic variation of Salmonella in host tissues delays eradication by antimicrobial chemotherapy. *Cell* **158**, 722–733 (2014). 25126781 10.1016/j.cell.2014.06.045

[39] Luk, C. H. et al. Salmonella enters a dormant state within human epithelial cells for persistent infection. *PLoS Pathog.* **17**, e1009550 (2021). 33930101 10.1371/journal.ppat.1009550 PMC8115778

[40] Irwin, S. M. et al. Bedaquiline and Pyrazinamide Treatment Responses Are Affected by Pulmonary Lesion Heterogeneity in Mycobacterium tuberculosis Infected C3HeB/FeJ Mice. *ACS Infect. Dis.* **2**, 251–267 (2016). 27227164 10.1021/acsinfecdis.5b00127 PMC4874602

[41] Driver, E. R. et al. Evaluation of a mouse model of necrotic granuloma formation using C3HeB/FeJ mice for testing of drugs against Mycobacterium tuberculosis. *Antimicrob. Agents Chemother.* **56**, 3181–3195 (2012). 22470120 10.1128/AAC.00217-12 PMC3370740

[42] Bankhead, P. et al. QuPath: Open source software for digital pathology image analysis. *Sci. Rep.* **7**, 16878 (2017). 29203879 10.1038/s41598-017-17204-5 PMC5715110

[43] Lanoix, J. P., Lenaerts, A. J. & Nuermberger, E. L. Heterogeneous disease progression and treatment response in a C3HeB/FeJ mouse model of tuberculosis. *Dis. Model Mech.* **8**, 603–610 (2015). 26035868 10.1242/dmm.019513 PMC4457036

[44] Huang, L., Nazarova, E. V., Tan, S., Liu, Y. & Russell, D. G. Growth of Mycobacterium tuberculosis in vivo segregates with host macrophage metabolism and ontogeny. *J. Exp. Med.* **215**, 1135–1152 (2018). 29500179 10.1084/jem.20172020 PMC5881470

[45] Amoura, A. et al. Variability in cell division among anatomical sites shapes Escherichia coli antibiotic survival in a urinary tract infection mouse model. *Cell Host Microbe* **32**, 900–912.e904 (2024). 38759643 10.1016/j.chom.2024.04.015

[46] Helaine, S., Conlon, B. P., Davis, K. M. & Russell, D. G. Host stress drives tolerance and persistence: The bane of anti-microbial therapeutics. *Cell Host Microbe* **32**, 852–862 (2024). 38870901 10.1016/j.chom.2024.04.019 PMC11446042

[47] Koch, A. & Wilkinson, R. J. The road to drug resistance in Mycobacterium tuberculosis. *Genome Biol.* **15**, 520 (2014). 25417849 10.1186/s13059-014-0520-1 PMC4282145

[48] Singh, R. et al. Recent updates on drug resistance in Mycobacterium tuberculosis. *J. Appl. Microbiol.* **128**, 1547–1567 (2020). 31595643 10.1111/jam.14478

[49] Lapinska, U. et al. Fast bacterial growth reduces antibiotic accumulation and efficacy. *Elife* **11**, 10.7554/eLife.74062 (2022). PMC9173744 35670099

[50] Miyahara, Y. et al. Survival of ampicillin-treated uropathogenic Escherichia coli is independent of single-cell growth rates. *NPJ Antimicrob. Resist* **4**, 7 (2026). 41629675 10.1038/s44259-025-00180-6 PMC12864937

[51] Bermudez, L. E. & Inderlied, C. B. Effect of Mycobacterium avium infection on the influx, accumulation, and efflux of KRM-1648 by human macrophages. *Micro. Drug Resist.* **3**, 277–282 (1997). 10.1089/mdr.1997.3.277 9270999

[52] Adams, R. A. et al. Rifamycin antibiotics and the mechanisms of their failure. *J. Antibiot. (Tokyo)* **74**, 786–798 (2021). 34400805 10.1038/s41429-021-00462-x

[53] Zhu, J. H. et al. Rifampicin can induce antibiotic tolerance in mycobacteria via paradoxical changes in rpoB transcription. *Nat. Commun.* **9**, 4218 (2018). 30310059 10.1038/s41467-018-06667-3 PMC6181997

[54] Sinclair, P., Carballo-Pacheco, M. & Allen, R. J. Growth-dependent drug susceptibility can prevent or enhance spatial expansion of a bacterial population. *Phys. Biol.* **16**, 046001 (2019). 30909169 10.1088/1478-3975/ab131e

[55] Karakousis, P. C., Williams, E. P. & Bishai, W. R. Altered expression of isoniazid-regulated genes in drug-treated dormant Mycobacterium tuberculosis. *J. Antimicrob. Chemother.* **61**, 323–331 (2008). 18156607 10.1093/jac/dkm485

[56] Ahmad, Z. et al. Biphasic kill curve of isoniazid reveals the presence of drug-tolerant, not drug-resistant, Mycobacterium tuberculosis in the guinea pig. *J. Infect. Dis.* **200**, 1136–1143 (2009). 19686043 10.1086/605605

[57] Ojkic, N., Serbanescu, D. & Banerjee, S. Antibiotic Resistance via Bacterial Cell Shape-Shifting. *mBio* **13**, e0065922 (2022). 35616332 10.1128/mbio.00659-22 PMC9239207

[58] Mishra, R. et al. Targeting redox heterogeneity to counteract drug tolerance in replicating Mycobacterium tuberculosis. *Sci. Transl. Med.* **11**, 10.1126/scitranslmed.aaw6635 (2019). PMC7212044 31723039

[59] Wakamoto, Y. et al. Dynamic persistence of antibiotic-stressed mycobacteria. *Science* **339**, 91–95 (2013). 23288538 10.1126/science.1229858

[60] Lamont, E. A., Dillon, N. A. & Baughn, A. D. The Bewildering Antitubercular Action of Pyrazinamide. *Microbiol. Mol. Biol. Rev.* **84**, 10.1128/MMBR.00070-19 (2020). PMC7062198 32132245

[61] Zhang, Y., Scorpio, A., Nikaido, H. & Sun, Z. Role of acid pH and deficient efflux of pyrazinoic acid in unique susceptibility of Mycobacterium tuberculosis to pyrazinamide. *J. Bacteriol.* **181**, 2044–2049 (1999). 10094680 10.1128/jb.181.7.2044-2049.1999 PMC93615

[62] Gopal, P. et al. Pyrazinoic Acid Inhibits Mycobacterial Coenzyme A Biosynthesis by Binding to Aspartate Decarboxylase PanD. *ACS Infect. Dis.* **3**, 807–819 (2017). 28991455 10.1021/acsinfecdis.7b00079 PMC5734868

[63] Campo-Perez, V., Cendra, M. D. M., Julian, E. & Torrents, E. Easily applicable modifications to electroporation conditions improve the transformation efficiency rates for rough morphotypes of fast-growing mycobacteria. *N. Biotechnol.* **63**, 10–18 (2021). 33636348 10.1016/j.nbt.2021.02.003

[64] Santucci, P. et al. Visualizing Pyrazinamide Action by Live Single-Cell Imaging of Phagosome Acidification and Mycobacterium tuberculosis pH Homeostasis. *mBio* **13**, e0011722 (2022). 35323041 10.1128/mbio.00117-22 PMC9040869

[65] Sharma, S. et al. Simple and rapid method to determine antimycobacterial potency of compounds by using autoluminescent Mycobacterium tuberculosis. *Antimicrob. Agents Chemother.* **58**, 5801–5808 (2014). 25049243 10.1128/AAC.03205-14 PMC4187967

[66] Hilsenstein, V., Albert, M. & Buckley, G. VolkerH/DaskFusion: v0.0.1 (v0.0.1). *Zenodo*10.5281/zenodo.10715850 (2022).

[67] Dask. *Dask: Library for dynamic task scheduling*, http://dask.pydata.org (2016).

[68] Pachitariu, M. & Stringer, C. Cellpose 2.0: how to train your own model. *Nat. Methods* **19**, 1634–1641 (2022). 36344832 10.1038/s41592-022-01663-4 PMC9718665

[69] Ulicna, K., Vallardi, G., Charras, G. & Lowe, A. R. Automated Deep Lineage Tree Analysis Using a Bayesian Single Cell Tracking Approach. *Frontiers in Computer Science* **3**, 10.3389/fcomp.2021.734559 (2021).

[70] Seabold, S. P., Josef. in *9th Python in Science Conference* (2010).

[71] Moore, J. et al. OME-Zarr: a cloud-optimized bioimaging file format with international community support. *Histochem Cell Biol.* **160**, 223–251 (2023). 37428210 10.1007/s00418-023-02209-1 PMC10492740

[72] Rocklin, M. in *Proceedings of the 14th Python in Science Conference*. 130–136.

[73] Waskom, M. seaborn: statistical data visualization. *Journal of Open Source Software* **6**, 10.21105/joss.03021 (2021).

[74] Day, N. macrohet: single-cell trajectory dataset of intracellular Mycobacterium tuberculosis population heterogeneity in iPSC-derived macrophages [Data set]. Zenodo. 10.5281/zenodo.20831464 (2026).

[75] Day, N. nthndy/macrohet: macrohet: Single-cell analysis of intracellular Mycobacterium tuberculosis population heterogeneity in iPSC-derived macrophages (v2.0.0). Zenodo. 10.5281/zenodo.20819399 (2026).

