# Supplementary material for: Fast-growing intracellular Mycobacterium tuberculosis populations evade antibiotic treatment
Source: Nat Commun. 2026 Jul 28;17:9164. doi: 10.1038/s41467-026-75915-8 (PMC13522540; doi:10.1038/s41467-026-75915-8)
Supplement: Supplementary file 1 [file 41467_2026_75915_MOESM1_ESM.pdf]

Supplementary Figure S1

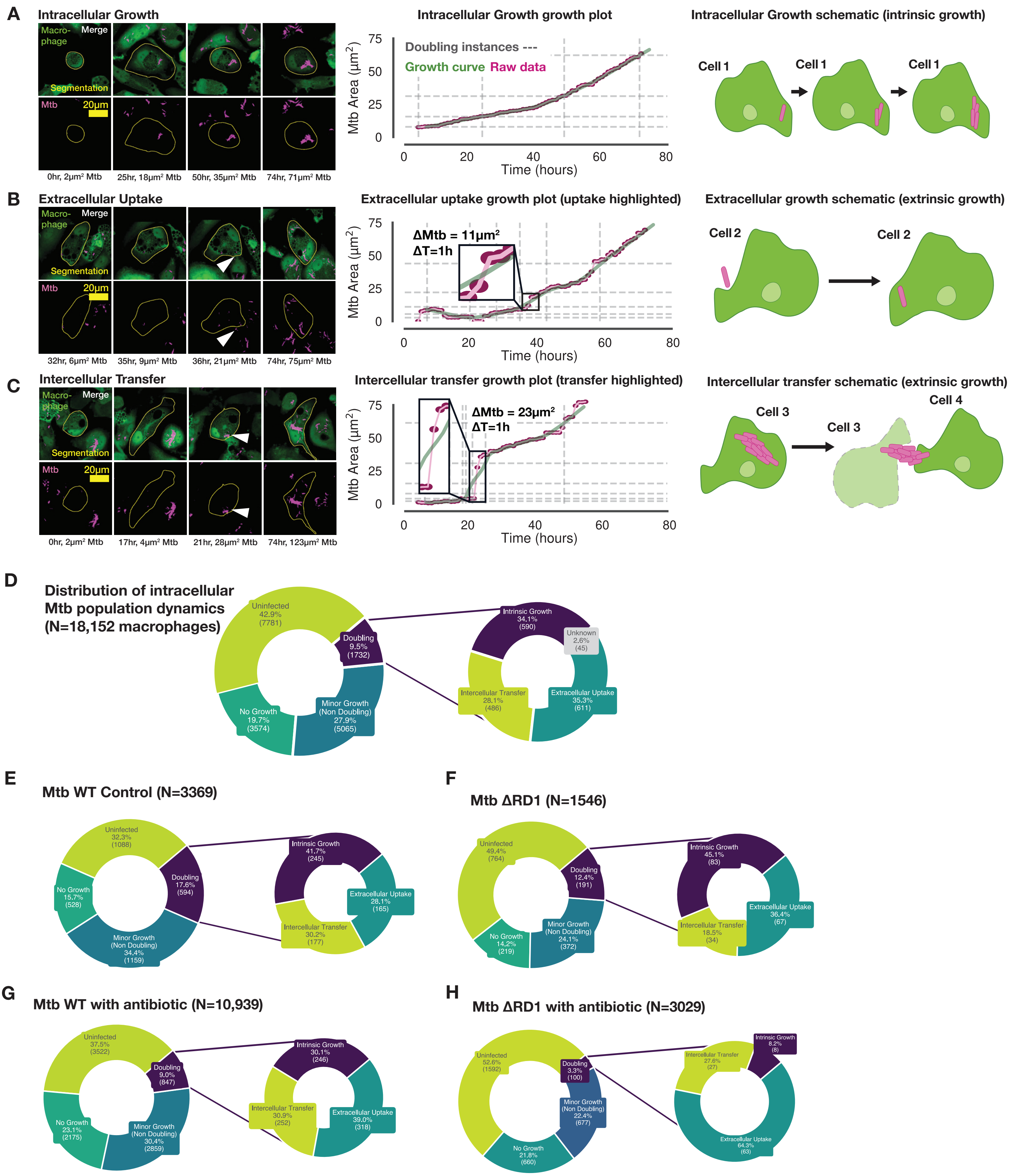

Supplementary Fig. S1. Classification and quantification of extracellular and intracellular Mtb growth dynamics *in cellulo*.

(A) Single-cell example of wholly intrinsic, intracellular Mtb growth resulting in typical doubling time (20 h) of intracellular Mtb. Representative images (left) with LOWESS growth curve in green, raw data in magenta, the doubling points in grey dotted lines (center) and illustrative diagram (right).

(B) Single-cell example of extrinsic, extracellular Mtb uptake (white arrow) resulting in rapid increase (11 μm^2 in 1 h) of intracellular Mtb from the perspective of the host macrophage. Representative images (left) with LOWESS growth curve in green, raw data in magenta, the doubling points in grey dotted lines (center) and illustrative diagram (right).

(C) Single-cell example of extrinsic, intercellular Mtb transfer (white arrow) resulting in rapid increase (23 μm^2 in 1 h) of intracellular Mtb from the perspective of the host macrophage. Representative images (left) with LOWESS growth curve in green, raw data in magenta, the doubling points in grey dotted lines (center) and illustrative diagram (right).

(D) Our analysis tracked a total of N_{cell} = 18,152 macrophages across all conditions. Of these, 42.9% (7,781) showed no detectable signs of intracellular infection, 19.7% (3,574) were infected with no measurable growth, 27.9% (5,065) were infected with minor growth insufficient for an intracellular population doubling, and 9.5% (1,732) of macrophages exhibited intracellular Mtb population doublings. Of these doublings, 34.1% (590) were attributable to intrinsic intracellular growth, whereas the remaining population doublings were due to intercellular transfer (28.1%), extracellular uptake (35.3%), or unknown (2.6%). Therefore, out of a possible 18,152 tracked macrophages, we focus on 590 that constituted the most permissive hosts.

(E) Our control analysis tracked N_{cell} = 3,369 untreated macrophages. Of these, 32.3% (1,088) showed no detectable signs of intracellular infection, 15.7% (528) were infected with no measurable growth, 34.4% (1,159) were infected with minor growth insufficient for an intracellular population doubling, and 17.6% (594) exhibited intracellular Mtb population doublings. Of these, 41.7% (245) were attributable to intrinsic intracellular growth, with the remainder due to intercellular transfer (30.2%) or extracellular uptake (28.1%). Therefore, we focus on the 245 macrophages that constituted the most permissive hosts in the control group.

(F) Our additional control analysis tracked N_{cell} = 1,546 untreated Mtb-ΔRD1-infected macrophages. Of these, 49.4% (764) showed no detectable signs of infection, 14.2% (219) were infected with no measurable growth, 24.1% (372) were infected with minor growth, and 12.4% (191) exhibited intracellular population doublings. Of these doublings, 45.1% (83) were attributable to intrinsic intracellular growth, whereas the remaining were due to intercellular transfer (18.5%) or extracellular uptake (36.4%). Therefore, we focus on the 83 macrophages that constituted the most permissive hosts for Mtb-ΔRD1.

(G) Our analysis tracked N_{cell} = 10,939 antibiotic-treated macrophages. Of these, 32.2% (3,522) showed no detectable signs of infection, 19.9% (2,175) were infected with no measurable growth, 26.1% (2,859) were infected with minor growth, and 7.7% (847) exhibited intracellular population doublings. Of these doublings, 30.1% (248) were attributable to intrinsic intracellular growth, with the remainder due to intercellular transfer (30.9%) or extracellular uptake (39.0%). Therefore, out of a possible 10,939 tracked macrophages, we focus on 248 that constituted the most permissive hosts under antibiotic pressure.

(H) Our additional control analysis tracked N_{cell} = 3,029 antibiotic-treated Mtb-ΔRD1-infected macrophages. Of these, 52.6% (1,592) showed no detectable signs of infection, 21.8% (660) were infected with no measurable growth, 22.4% (677) were infected with minor growth, and 3.3% (100) exhibited intracellular population doublings. Of these doublings, only 8.2% (8) were attributable to intrinsic intracellular growth, whereas the majority were due to extracellular uptake (64.3%) or intercellular transfer (27.6%). Therefore, out of a possible 3,029 tracked macrophages, only 8 constituted the most permissive hosts, precluding further meaningful analysis of doubling times in this condition.

Supplementary Figure S2

A **Intracellular Mtb doubling times versus initial bacterial area (averaged 5h) in iPSDM**

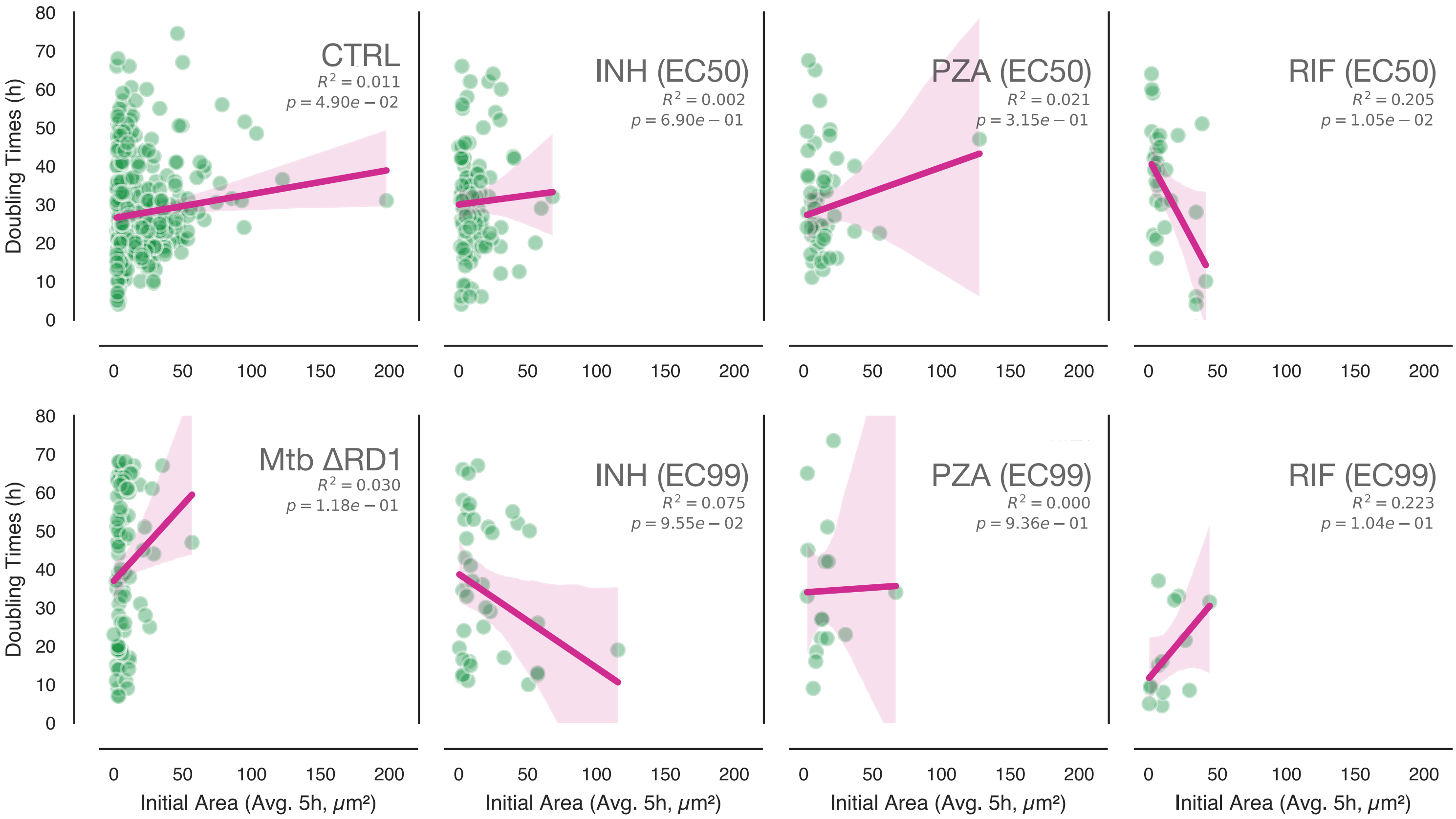

B

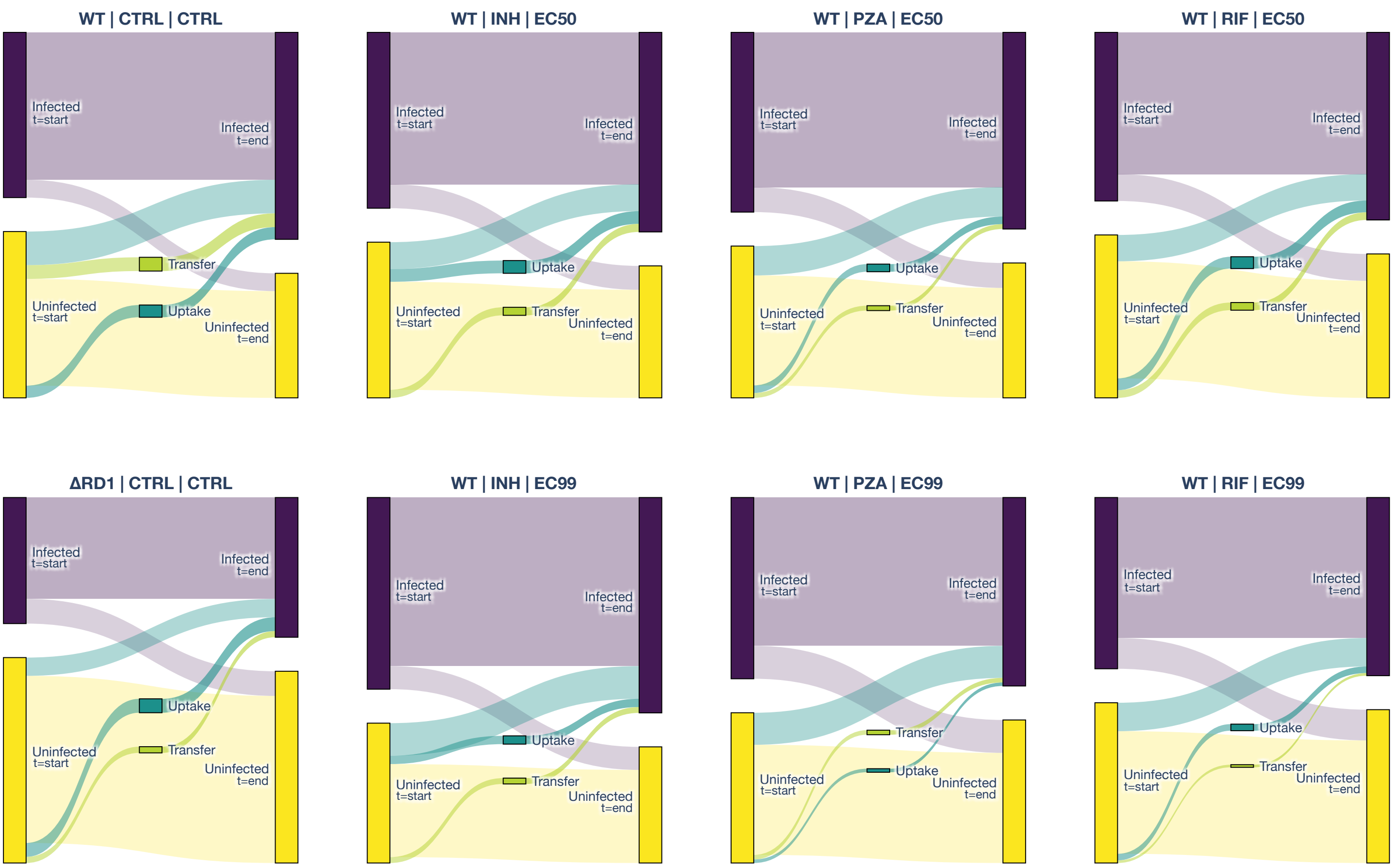

Supplementary Fig. S2. Independence of replication rate from initial burden and visualization of macrophage infection dynamics.

(A) Correlation analysis between intracellular Mtb replication rate (doubling time) and initial bacterial burden (measured as mean bacterial area over the first 5 h of tracking, μm^2). Data are shown for Mtb WT (control and antibiotic-treated) and ΔRD1 strains. The magenta line represents the linear regression fit with 95% confidence intervals (shaded region). The coefficient of determination (R^2) and p-values are displayed for each condition. The consistently low R^2 values indicate that intracellular growth rates are independent of the initial bacterial load within the macrophage.

(B) Sankey diagrams visualizing macrophage fate and infection dynamics across the duration of the time-lapse acquisition. The left nodes represent the infection status at the start of tracking (t_{start}), and the right nodes represent the status at the end (t_{end}). Purple indicates infected macrophages; yellow indicates uninfected macrophages. Flows represent the continuity or change in state, highlighting extrinsic infection events, specifically extracellular uptake and intercellular transfer, which were excluded from the intrinsic growth rate analysis. Diagrams are separated by bacterial strain and antibiotic treatment condition (EC50 and EC99).

Supplementary Figure S3

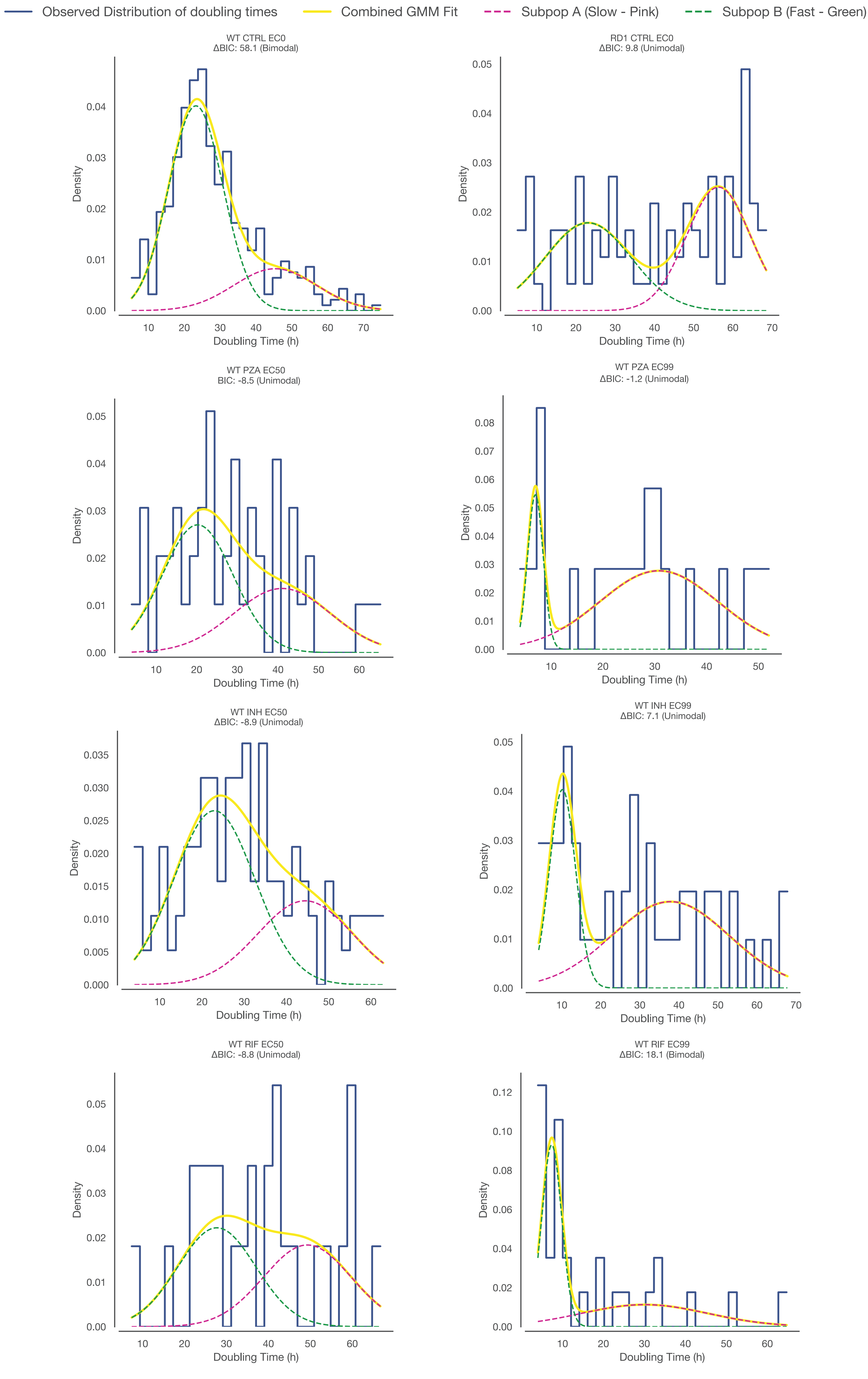

Supplementary Fig. S3. Gaussian Mixture Model (GMM) decomposition of intracellular doubling time heterogeneity.

Histograms displaying the probability density of single-cell doubling times across Mtb strains and antibiotic treatment conditions. The observed data (blue step line) were analyzed using a Gaussian Mixture Model to resolve underlying phenotypic subpopulations. The yellow solid line represents the combined best-fit model. This global fit is deconstructed into two distinct Gaussian components: a fast-growing subpopulation (green dashed line, centered on lower doubling times) and a slow-growing subpopulation (pink dashed line, centered on higher doubling times). The *ΔBIC* score (difference in Bayesian Information Criterion) is displayed for each condition; high positive values indicate that the data are better described by a 2-component Gaussian mixture rather than a single distribution, though this does not exclude alternative explanations such as heavy-tailed unimodal distribution. Note that while untreated Wild-Type (WT CTRL) and Rifampicin-treated (WT RIF EC99) populations are better described by a 2-component mixture with a preserved fast-growing peak, other conditions (such as ΔRD1) are better described by a single, slower distribution.

Supplementary Figure S4

A **Fold change in total wild-type Mtb area across entire field of view**
Each well shown as a distinct green line with the ensemble average shown in magenta

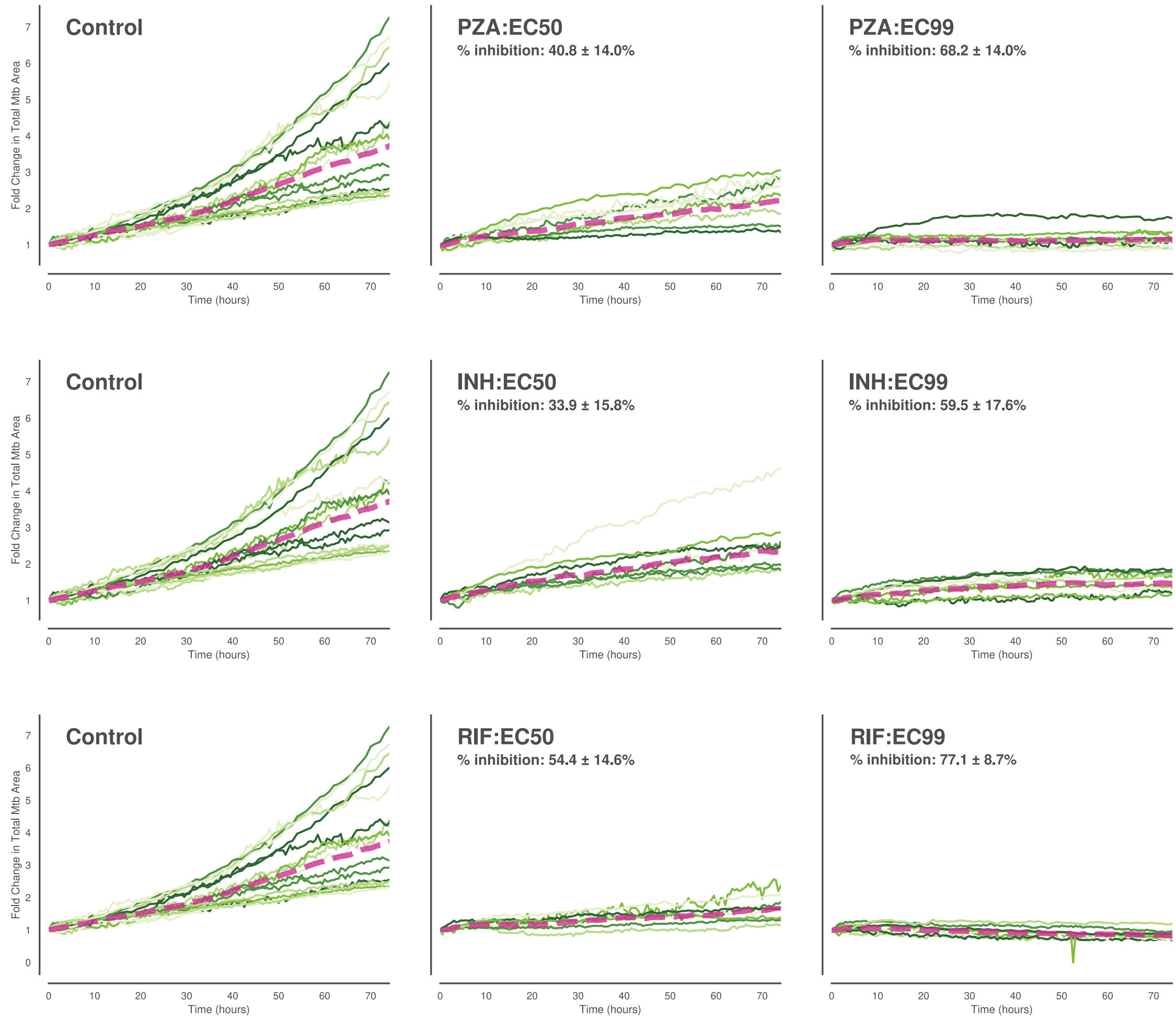

B **Fold change in total Mtb ΔRD1 area across entire field of view**
Each well shown as a distinct green line with the ensemble average shown in magenta

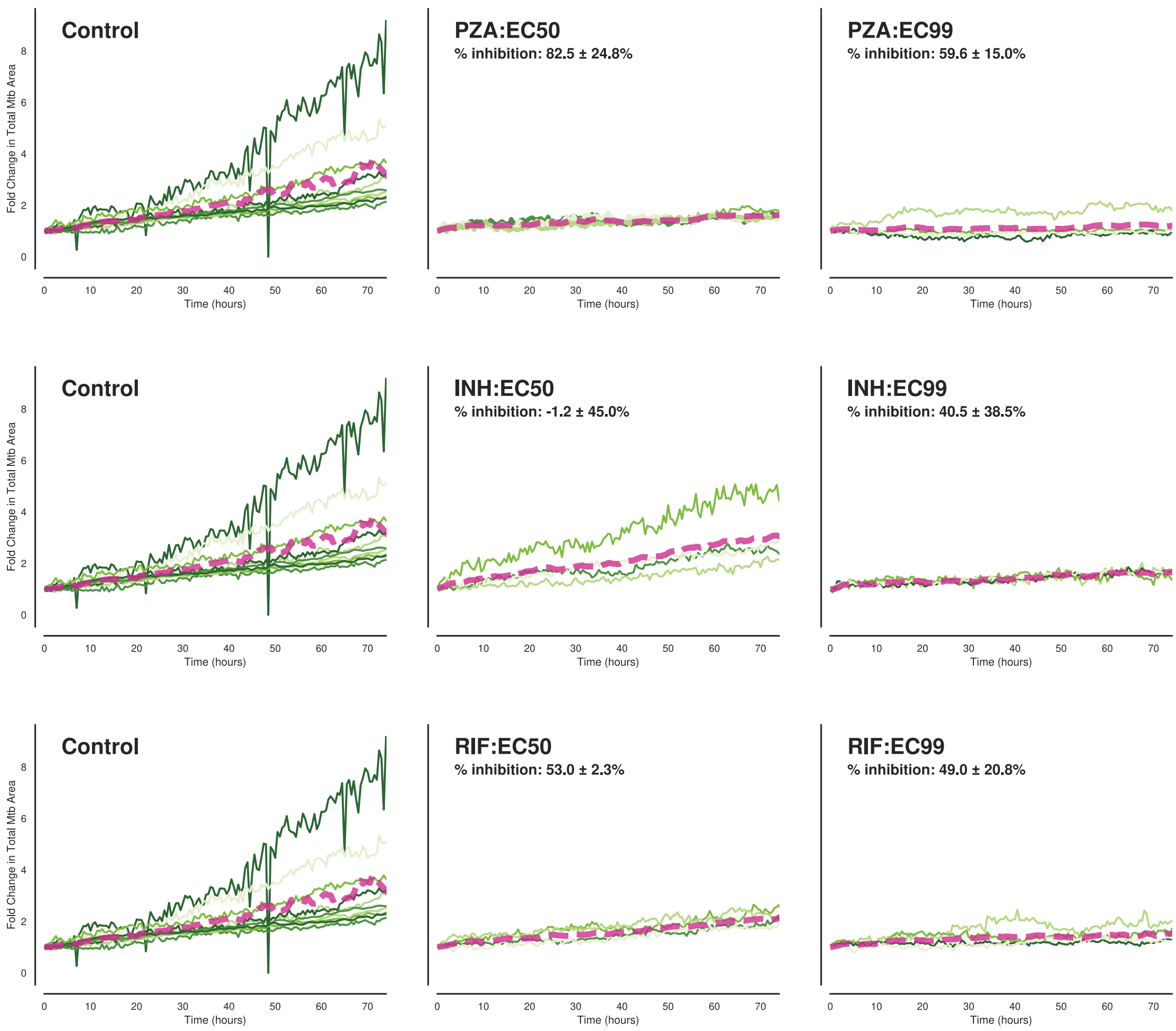

Supplementary Fig. S4. Population-level quantification of intracellular Mtb growth kinetics and antibiotic inhibition.

(A) Longitudinal analysis of intracellular Mtb Wild-Type (WT) growth burden, measured as the fold change in total fluorescent bacterial area per well relative to $t = 0$. Macrophages were treated with Pyrazinamide (PZA), Isoniazid (INH), or Rifampicin (RIF) at concentrations corresponding to EC50 and EC99. Thin green lines represent individual imaging wells (technical replicates), illustrating well-to-well variability. The thick magenta dashed line represents the ensemble mean across all wells. Inset values indicate the percentage of growth inhibition relative to the untreated control (Mean ± SD), confirming the bulk efficacy of the antibiotic treatments.

(B) Equivalent growth kinetic analysis for the attenuated Mtb ΔRD1 strain under identical control and antibiotic-treated conditions. Note the generally slower growth trajectory of the control ΔRD1 strain compared to WT, and the corresponding inhibition profiles.

Supplementary Figure S5

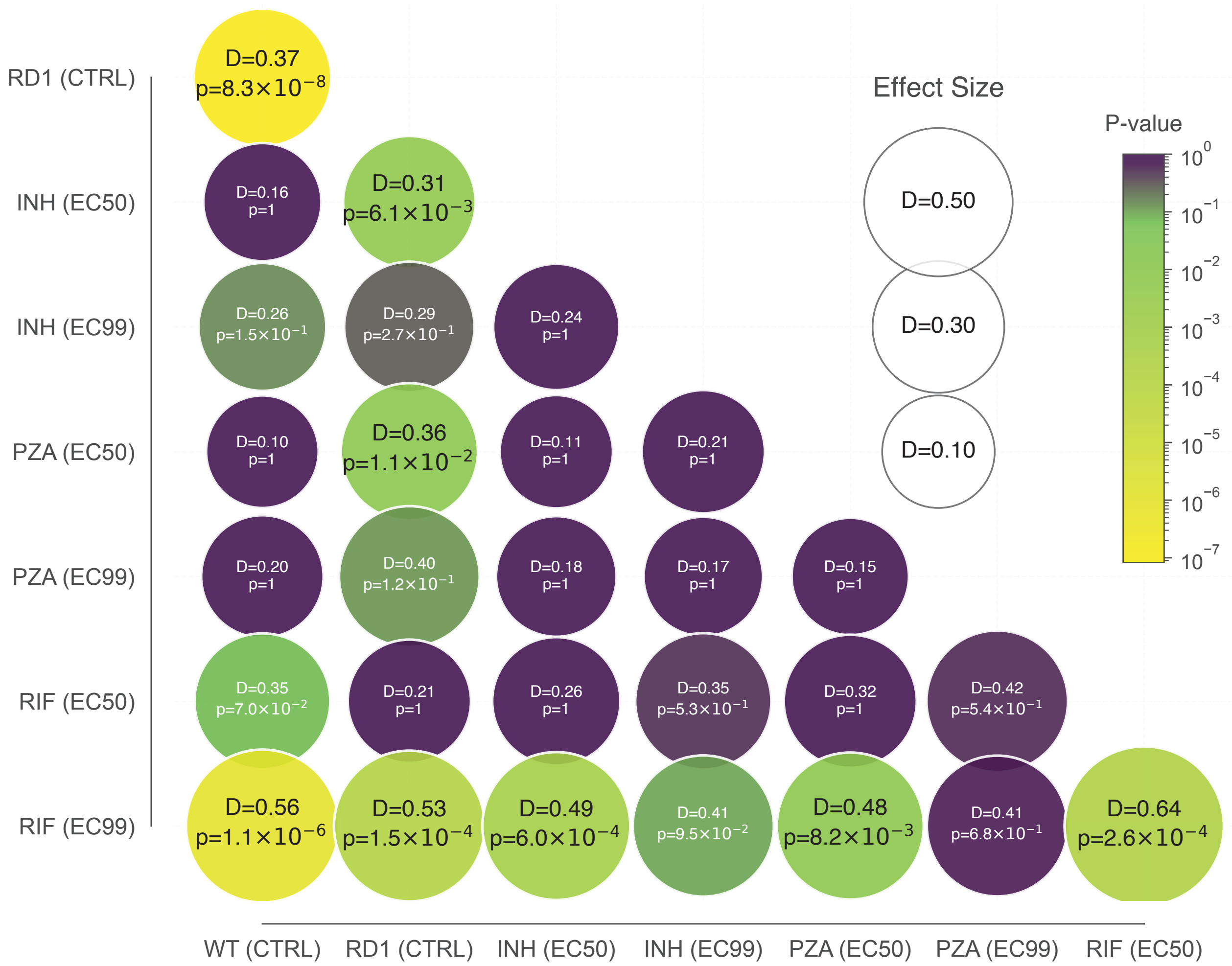

Supplementary Fig. S5. Pairwise statistical comparison of intracellular doubling time distributions. Matrix visualization of the Kolmogorov-Smirnov (KS) test statistics comparing the shape and location of doubling time distributions across all experimental conditions. The color of each node represents the statistical significance (P-value) of the difference, ranging from dark purple (non-significant, $p > 0.05$) to bright yellow (highly significant, $p < 10^{-6}$). The size of each circle is proportional to the KS effect size (D statistic), which quantifies the maximum distance between the two cumulative distribution functions. Larger circles indicate a greater magnitude of phenotypic divergence. The specific D statistic and p-value are listed within each node.

Supplementary Figure S6

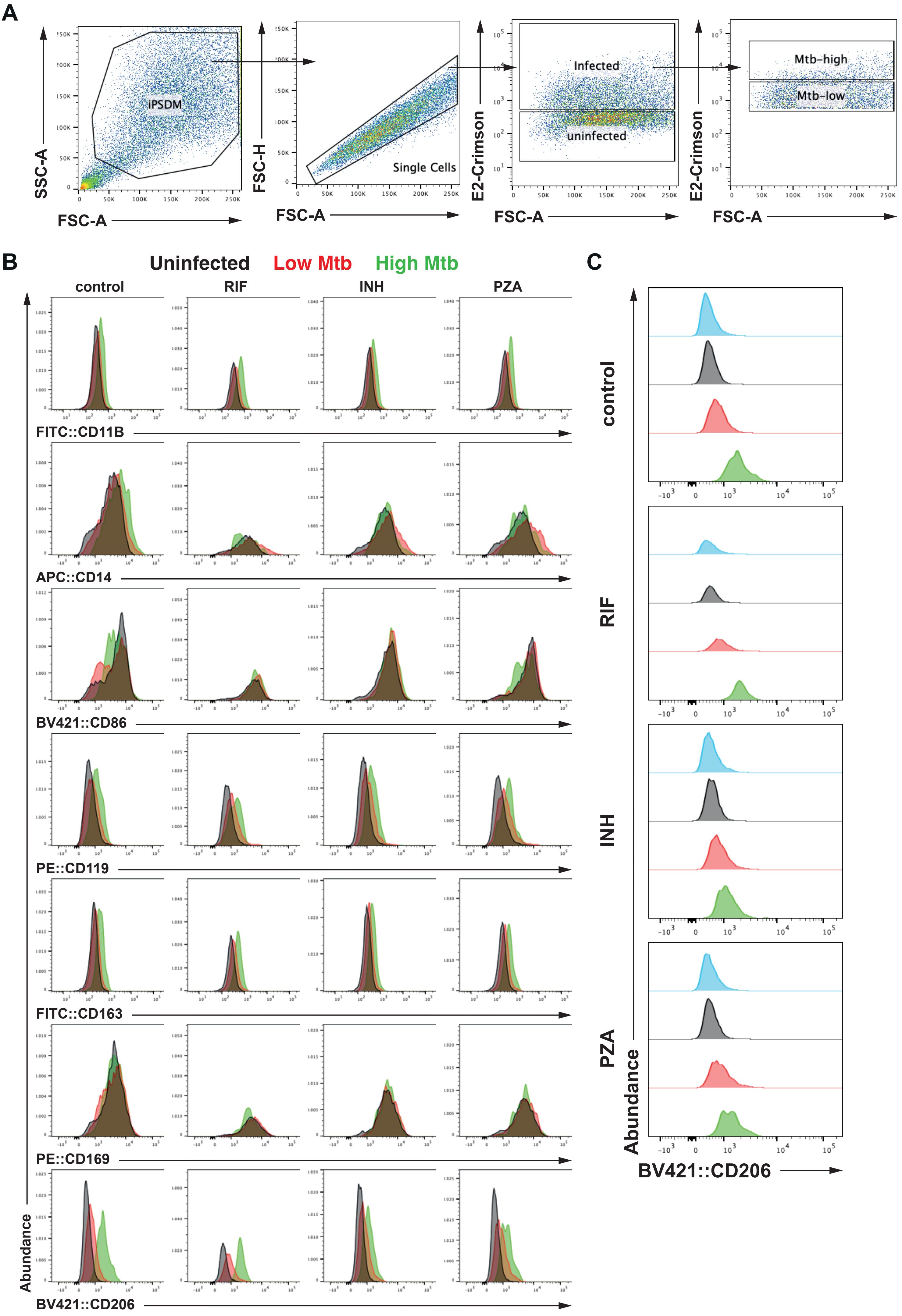

Supplementary Fig. S6. Surface marker characterization of Mtb-infected iPSDM.
(A) Gating strategy of Mtb-infected iPSDM.
(B) Expression of CD11B, CD14, CD86, CD119, CD163, CD169, CD206 in Mtb-infected iPSDM measured by flow cytometry, representative plots showing uninfected (black), Mtb-low (red) and Mtb-high (green) iPSDM samples. (*n* = 3 independent experiments).
(C) Expression of CD206 in uninfected iPSDM (cyan) and Mtb-infected iPSDM measured by flow cytometry, representative plots showing uninfected (black), Mtb-low (red) and Mtb-high (green) iPSDM samples. (*n* = 3 independent experiments).

Supplementary Figure S7

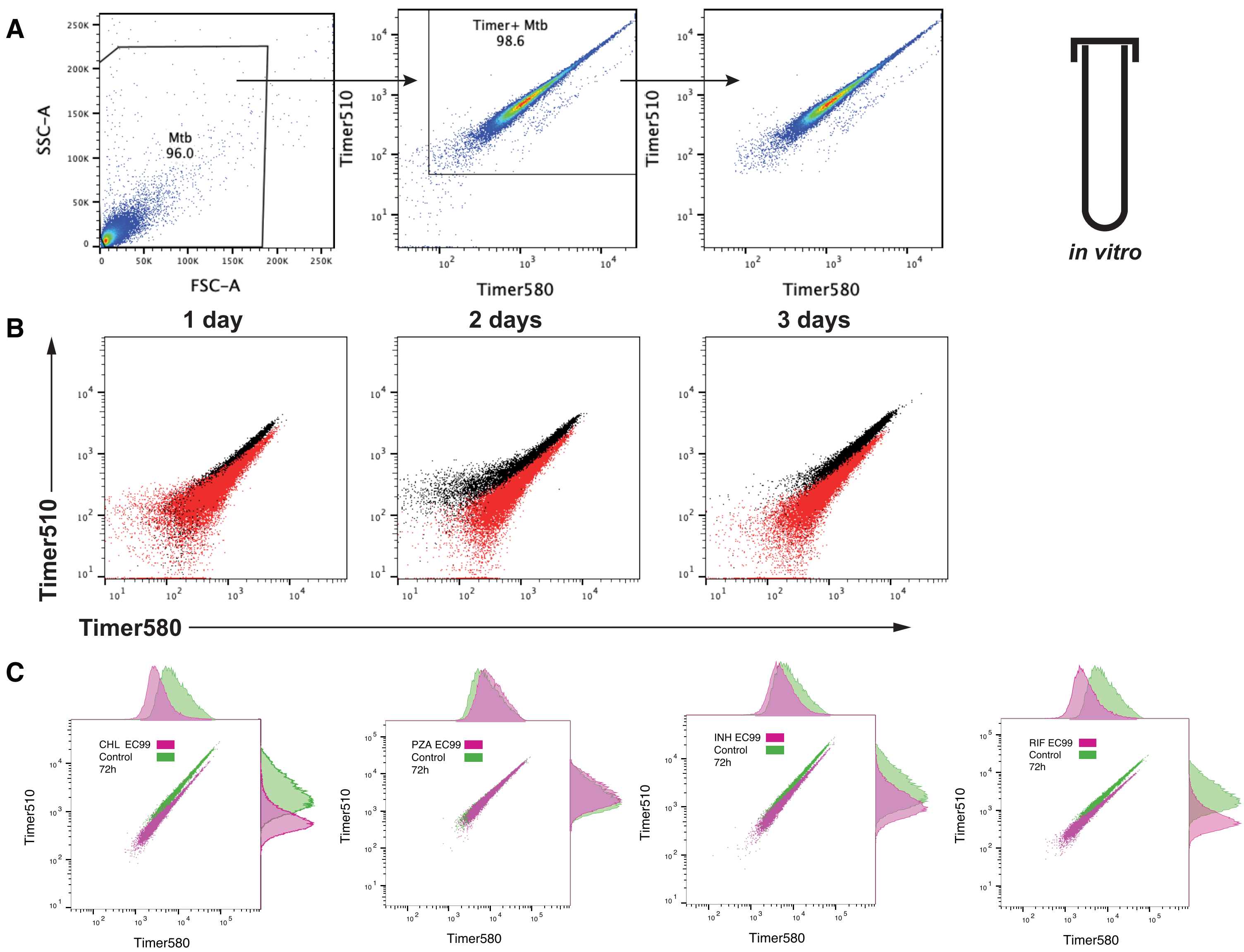

Supplementary Fig. S7. Flow cytometry validation and characterization of the Mtb-Timer replication reporter *in vitro*.

(A) Gating strategy of Mtb-Timer. Bacteria were first gated from the SSC-A vs FSC-A plot to remove bacterial clumps; Mtb-Timer bacteria were then gated from the Mtb population. Representative data from one of three independent experiments (n = 3 independent experiments).

(B) Timer profiles of Mtb treated with chloramphenicol *in vitro* for 1, 2 and 3 days, representative plots showing untreated control (black) and chloramphenicol treated (red) samples. (n = 3 independent experiments).

(C) Timer profiles of Mtb treated with antibiotic *in vitro* for 3 days. Representative data of one out of the three independent experiments (n = 3 independent experiments).

Supplementary Figure S8

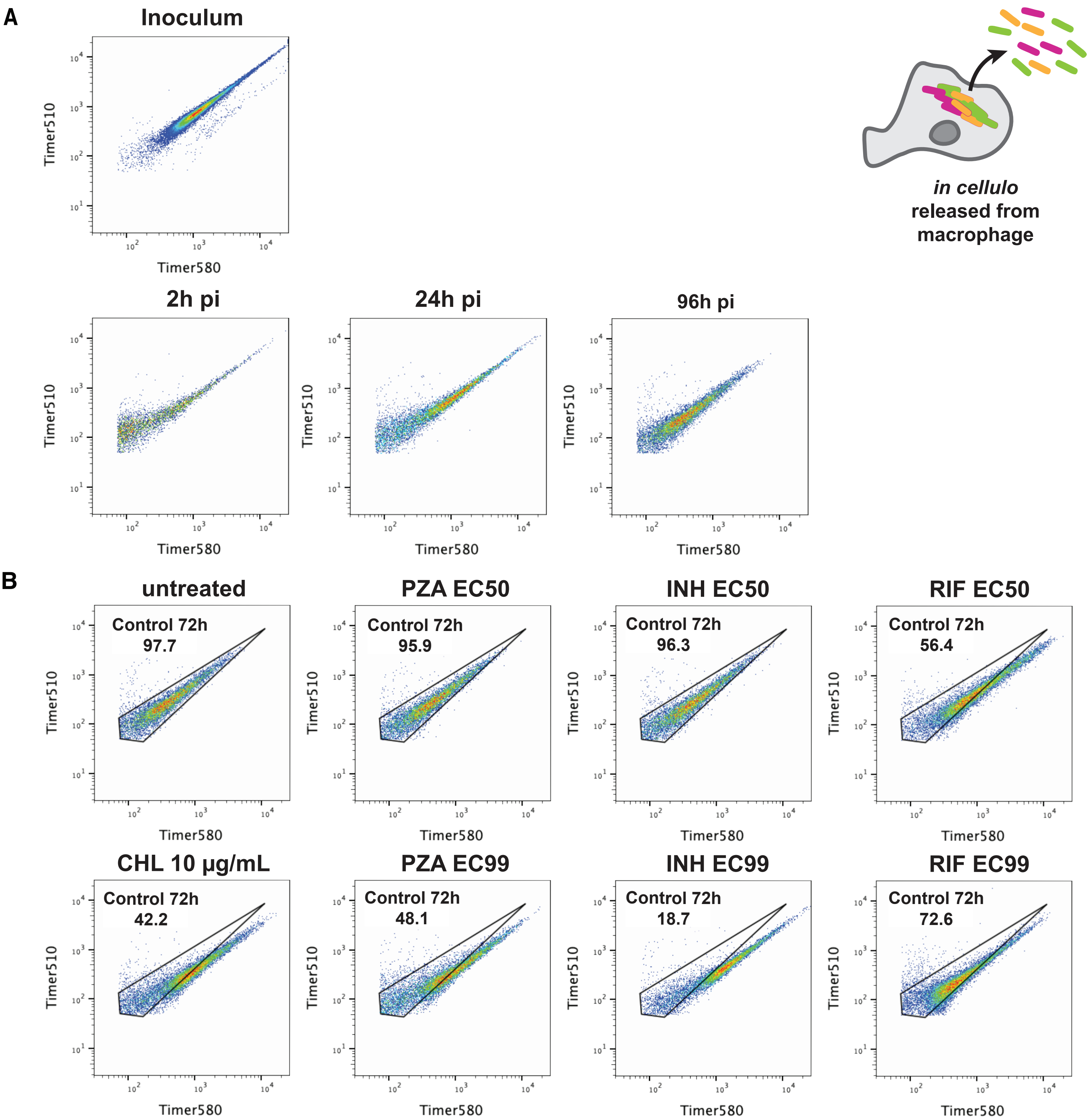

Supplementary Fig. S8. Flow cytometry validation and characterization of the Mtb-Timer replication reporter *in cellulo*.

(A) Mtb-Timer profiles of inoculum and *in cellulo* Mtb at 2 h, 24 h and 96 h after infection. Representative data of one out of the three independent experiments ($n = 3$ independent experiments).

(B) Mtb-Timer profiles of *in cellulo* Mtb at 96 h after infection under antibiotic treatment. Gate "Control 72 h" is gated from the control condition to depict the Mtb-Timer profile without antibiotic treatment. Representative data of one out of three independent experiments ($n = 3$ independent experiments).

Supplementary Figure S9

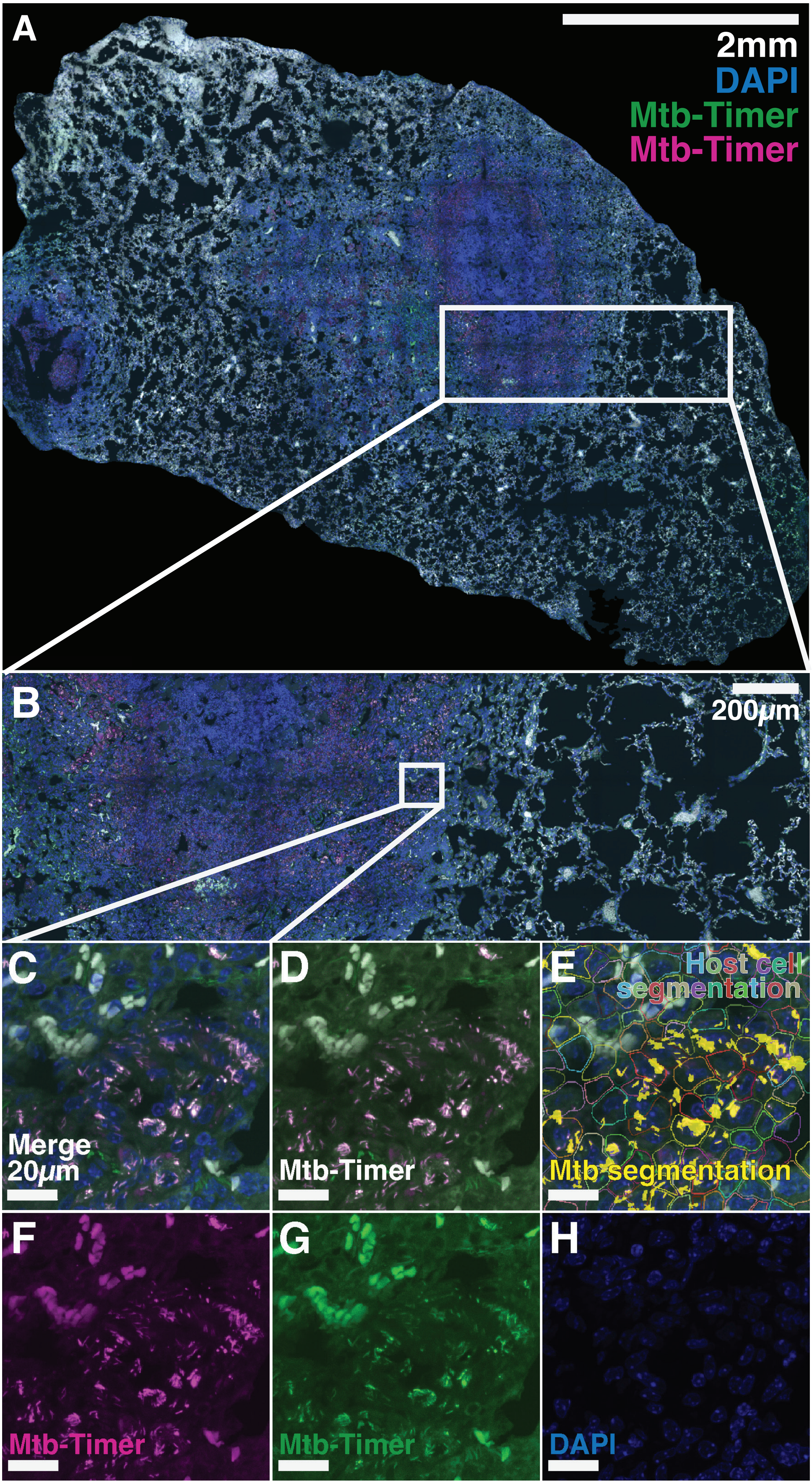

Supplementary Fig. S9. Multi-scale confocal imaging and single-cell segmentation pipeline in Mtb-Timer infected mouse lungs.
(A-B) Large-scale tiled scan of a whole lung lobectomy **(A)** and a magnified view of a lesion region **(B)**, visualizing the distribution of Mtb-Timer (green and magenta) relative to host nuclei (DAPI, blue).
(C-H) High-resolution details illustrating the segmentation strategy. A representative field of view (**C**, composite) is split into component channels: combined Mtb-Timer fluorescence **(D)**, mature red Mtb-Timer **(F)**, newly synthesized green Mtb-Timer **(G)**, and host nuclei **(H)**. These signals are used to generate segmentation masks **(E)**, with the manually identified individual bacterial units (yellow outlines) and automatically segmented host cell boundaries (colored lines).

Supplementary Tables

Supplementary Table S1: Summary statistics for phenotypic heterogeneity across experimental conditions.

Condition	Mean	Standard Deviation	*CV*	N_{dt}	Description
WT (CTRL)	28.48	12.81	0.45	402	Moderate Heterogeneity
RD1 (CTRL)	40.09	19.31	0.48	87	Moderate Heterogeneity
PZA (EC50)	28.70	14.57	0.51	48	High Heterogeneity
PZA (EC99)	25.88	14.35	0.55	22	High Heterogeneity
INH (EC50)	30.67	14.62	0.48	97	Moderate Heterogeneity
INH (EC99)	28.48	18.21	0.64	48	High Heterogeneity
RIF (EC50)	38.09	14.89	0.39	28	Moderate Heterogeneity
RIF (EC99)	17.24	15.51	0.90	28	Extreme Heterogeneity

Supplementary Table S2: Fisher's Exact Test results for phenotypic shifts relative to wild-type control, with FDR-adjusted p-values.

Condition	FDR adjusted P-values	Significant
WT RIF EC99	2.42×10^{-7}	True
WT INH EC99	1.42×10^{-3}	True
WT PZA EC99	0.233	False
WT PZA EC50	0.242	False
WT RIF EC50	0.513	False
WT INH EC50	0.826	False
RD1 CTRL EC0	1.000	False

Supplementary Table S3: One-way ANOVA followed by Tukey's HSD post-hoc test was performed to compare the bacterial load (log_{10} CFU) in the lungs of C3HeB/FeJ mice across treatment groups (**Fig. 4E**).

Comparison (Group 1 vs. Group 2)	Mean Difference (log10 CFU)	95% Confidence Interval	Adjusted P-Value	Significance
PZA vs. Vehicle	1.99	[0.94, 3.06]	0.0002	*** (Significant)
RIF vs. Vehicle	1.38	[0.32, 2.44]	0.0090	** (Significant)
PZA vs. RIF	0.62	[-0.44, 1.67]	0.3295	ns (Not Significant)

Supplementary Table S4: Welch's ANOVA followed by Games-Howell post-hoc tests were performed to compare the mean single-cell Mtb-Timer ratio across treatment groups in C3HeB/FeJ mice (**Fig. 4F**). This method was selected to account for the unequal variances observed between the PZA and vehicle treatments.

**Comparison**	**Difference in Means**	**Standard Error**	**t-Statistic**	**P-Value**	**Significance**
**Global ANOVA**	—	—	$F = 4.07$	0.0381	* (Significant)
**PZA vs. Vehicle**	+1.28	0.52	2.45	0.0442	* (Significant)
**PZA vs. RIF**	+1.19	0.50	2.38	0.0466	* (Significant)
**RIF vs. Vehicle**	+0.09	0.17	0.49	0.9716	ns (Not Significant)
