# Supplementary material for: Fast-growing intracellular Mycobacterium tuberculosis populations evade antibiotic treatment
Source: Nat Commun. 2026 Jul 28;17:9164. doi: 10.1038/s41467-026-75915-8 (PMC13522540; doi:10.1038/s41467-026-75915-8)
Supplement: Supplementary file 2 [file 41467_2026_75915_MOESM2_ESM.pdf]

## Description of Additional Supplementary Files:

**Supplementary Movie 1:** High-content imaging pipeline tracking Mtb replication in human iPSC-derived macrophages. The left panel shows the merged 2-channel image data, with macrophages expressing EGFP in green and Mtb expressing E2-Crimson pseudo-colored magenta. The middle panel shows the same images with the segmentation overlaid in yellow. The right panel shows the tracked centroid location of each macrophage as a cyan line.

**Supplementary Movie 2:** Single-cell glimpse example of intracellular Mtb growth dynamics exhibiting normal (20-24 hours), slow (40 hours) and fast (14 hours) doubling times. The accompanying doubling time plot shows the intracellular Mtb burden over time as a magenta line, the LOWESS growth model as a green line and the subsequent doubling times and amounts as grey dashed lines.

**Supplementary Movie 3:** Single-cell glimpse example of wholly intrinsic intracellular Mtb growth dynamics, extracellular Mtb uptake and intercellular transfer of Mtb from a neighbouring macrophage. The accompanying doubling time plot shows the intracellular Mtb burden over time as a magenta line, the LOWESS growth model as a green line and the subsequent doubling times and amounts as grey dashed lines. The top example exhibits no sudden jumps in intracellular burden attributable to extrinsic Mtb populations. The middle example exhibits a minor jump in intracellular burden around 33 hours that is attributable to extracellular uptake of a neighboring population of Mtb. The lower example exhibits a major jump in intracellular burden around 13 hours that is attributable to intercellular transfer of a neighboring intracellular population of Mtb.
