# Supplementary material for: Fast-growing intracellular Mycobacterium tuberculosis populations evade antibiotic treatment
Source: Nat Commun. 2026 Jul 28;17:9164. doi: 10.1038/s41467-026-75915-8 (PMC13522540; doi:10.1038/s41467-026-75915-8)
Supplement: Supplementary file 3 [file 41467_2026_75915_MOESM6_ESM.pdf]

nature portfolio

Corresponding author(s): Maximiliano Gutierrez

Last updated by author(s): 3/6/2026

Reporting Summary

Nature Portfolio wishes to improve the reproducibility of the work that we publish. This form provides structure for consistency and transparency in reporting. For further information on Nature Portfolio policies, see our Editorial Policies and the Editorial Policy Checklist.

Statistics

For all statistical analyses, confirm that the following items are present in the figure legend, table legend, main text, or Methods section.

n/a	Confirmed	
☐	☒	The exact sample size (*n*) for each experimental group/condition, given as a discrete number and unit of measurement
☐	☒	A statement on whether measurements were taken from distinct samples or whether the same sample was measured repeatedly
☐	☒	The statistical test(s) used AND whether they are one- or two-sided<br>*Only common tests should be described solely by name; describe more complex techniques in the Methods section.*
☐	☒	A description of all covariates tested
☐	☒	A description of any assumptions or corrections, such as tests of normality and adjustment for multiple comparisons
☐	☒	A full description of the statistical parameters including central tendency (e.g. means) or other basic estimates (e.g. regression coefficient) AND variation (e.g. standard deviation) or associated estimates of uncertainty (e.g. confidence intervals)
☐	☒	For null hypothesis testing, the test statistic (e.g. *F*, *t*, *r*) with confidence intervals, effect sizes, degrees of freedom and *P* value noted<br>*Give P values as exact values whenever suitable.*
☒	☐	For Bayesian analysis, information on the choice of priors and Markov chain Monte Carlo settings
☐	☒	For hierarchical and complex designs, identification of the appropriate level for tests and full reporting of outcomes
☐	☒	Estimates of effect sizes (e.g. Cohen's *d*, Pearson's *r*), indicating how they were calculated

Our web collection on statistics for biologists contains articles on many of the points above.

Software and code

Policy information about availability of computer code

Data collection	Harmony software (Perkin Elmer, version 4.9) as a part of the Opera Phenix high-throughput microscopy acquisition.<br>Olympus cellSens Dimension version 4.1 as a part of the Olympus CSU-W1 SoRa Spinning Disk.<br>BD FACSDiva Software v8.0.1 for flow cytometry acquisition on the BD LSRFortessa (BD Biosciences).<br>QuPath version 0.51 for the loading and semi-automated annotation of mouse lung slices images from Olympus CSU-W1 SoRa Spinning Disk acquisition.
Data analysis	Bespoke Python (v3.10.19) analysis pipelines were used for image stitching, segmentation (custom-trained Cellpose v2 model), macrophage tracking (btrack v0.7.0) and doubling-time computation (LOWESS fitting). All scripts and documentation are openly available at https://github.com/nthndy/macrohet (v2.0.0).<br><br>Software and packages: Python 3.10.19; Cellpose v2; btrack 0.7.0; napari 0.5.6; Dask 2026.1.2; zarr 2.18.3; scikit-image 0.25.2; SciPy 1.15.2; scikit-learn 1.7.2; statsmodels 0.14.6; pandas 2.3.3; NumPy 2.2.6; seaborn 0.13.2; Matplotlib 3.10.8; tifffile 2025.5.10; QuPath 0.5.1; FlowJo 10.10.0.

For manuscripts utilizing custom algorithms or software that are central to the research but not yet described in published literature, software must be made available to editors and reviewers. We strongly encourage code deposition in a community repository (e.g. GitHub). See the Nature Portfolio guidelines for submitting code & software for further information.

Data

Policy information about availability of data

All manuscripts must include a data availability statement. This statement should provide the following information, where applicable:

- Accession codes, unique identifiers, or web links for publicly available datasets
- A description of any restrictions on data availability
- For clinical datasets or third party data, please ensure that the statement adheres to our policy

The processed single-cell trajectory dataset generated in this study (singlecell_trajectories_dataframe.parquet) is available in Zenodo under DOI 10.5281/zenodo.20831464 (https://doi.org/10.5281/zenodo.20831464). A downscaled example imaging acquisition sufficient to run the analysis pipeline is included in the code repository (https://github.com/nthndy/macrohet). The raw imaging data (~35 TB), in OME-NGFF (Zarr) format, are not deposited in a public repository owing to their size and are available under restricted access; requests should be directed to the corresponding author and will be answered within [X weeks], with data shared by [transfer method], and retained for at least [X years] after publication. All custom code is available at https://github.com/nthndy/macrohet. Source data are provided with this paper. All other data supporting the findings are included in the article and its Supplementary Information.

Research involving human participants, their data, or biological material

Policy information about studies with human participants or human data. See also policy information about sex, gender (identity/presentation), and sexual orientation and race, ethnicity and racism.

Reporting on sex and gender	This study did not involve human participants. iPSC-derived macrophages were generated from the KOLF2-C1 AAVS1 cell line, which is of male genetic sex. No sex- or gender-based analysis was performed, as this was a single-cell line model system.
Reporting on race, ethnicity, or other socially relevant groupings	This study did not involve human participants or collect race, ethnicity, or socially relevant variables. The iPSC line used (KOLF2-C1 AAVS1) is a reference line commonly used in genetic and stem cell research.
Population characteristics	See above. The study was performed using macrophages derived from a single, male-donor iPSC line (KOLF2-C1 AAVS1). No other human population-level information was applicable.
Recruitment	No human participants were recruited for this study. The iPSC line used was obtained as a gift from the Skarnes Lab (The Jackson Laboratory) and has been previously published and validated.
Ethics oversight	The use of the KOLF2-C1 AAVS1 human iPSC line followed institutional guidelines at the Francis Crick Institute. No new human samples or donor-derived materials were collected for this study.

Note that full information on the approval of the study protocol must also be provided in the manuscript.

Field-specific reporting

Please select the one below that is the best fit for your research. If you are not sure, read the appropriate sections before making your selection.

[x] Life sciences   [ ] Behavioural & social sciences   [ ] Ecological, evolutionary & environmental sciences

For a reference copy of the document with all sections, see nature.com/documents/nr-reporting-summary-flat.pdf

Life sciences study design

All studies must disclose on these points even when the disclosure is negative.

Sample size	Sample sizes were determined empirically based on previous imaging studies of Mycobacterium tuberculosis (Mtb) infection dynamics and pilot experiments establishing the variability of intracellular replication rates. For live-cell imaging, we tracked >20,000 single macrophages across four independent biological replicates and 121 fields of view, of which >940 contained quantifiable proliferative intracellular Mtb populations and were used for single-cell doubling-time analysis. For the in vivo experiments, 9 mice per condition (vehicle, rifampicin, PZA) were analysed across two independent batches. For flow cytometry, at least 10,000 single-bacterium events were recorded per condition. No formal power calculation was performed; the sample sizes achieved sufficient statistical power to detect differences in growth rate distributions and antibiotic responses using non-parametric tests.
Data exclusions	Exclusions were pre-defined based on segmentation and tracking confidence. Specifically, macrophages with non-doubling or unreliable Mtb trajectories (e.g. caused by intercellular transfer or tracking errors with poor LOWESS fit, R^2 threshold) were excluded from doubling time analysis. The total number of excluded cells and the criteria are detailed in Supplementary Figure S1. No data were excluded post hoc.
Replication	All key findings were replicated in at least four independent biological experiments using human iPSC-derived macrophages. Flow cytometry analyses of TimerMtb reporter strains were performed in three independent experiments. In vivo PZA and RIF treatment findings were confirmed across multiple animals, with single-cell resolution data provided for each animal.
Randomization	Samples were not randomized, as all macrophages were derived from a uniform iPSC line and plated evenly across wells. Drug treatments and bacterial strains were assigned to wells using a pre-determined plate layout to control for positional biases. Imaging and analysis were conducted identically across conditions.
Blinding	Blinding was not performed during live-cell image acquisition, as antibiotic treatment conditions and bacterial strains were encoded into the

experimental layout. However, phenotype classification (e.g. distinguishing intrinsic vs extrinsic growth) was conducted using a human-in-the-loop pipeline with blinded visual inspection to minimize bias.

Reporting for specific materials, systems and methods

We require information from authors about some types of materials, experimental systems and methods used in many studies. Here, indicate whether each material, system or method listed is relevant to your study. If you are not sure if a list item applies to your research, read the appropriate section before selecting a response.

Materials & experimental systems

n/a	Involved in the study
☐	☒ Antibodies
☐	☒ Eukaryotic cell lines
☒	☐ Palaeontology and archaeology
☐	☒ Animals and other organisms
☒	☐ Clinical data
☒	☐ Dual use research of concern
☒	☐ Plants

Methods

n/a	Involved in the study
☒	☐ ChIP-seq
☐	☒ Flow cytometry
☒	☐ MRI-based neuroimaging

Antibodies

Antibodies used	Anti-human surface markers (all 1:20): CD11b–FITC (Cat# 14-0112-82); CD14–APC (Cat# 555399); CD86–BV421 (Cat# 562433); CD119–PE (Cat# 558934); CD163–FITC (Cat# 563697); CD169–PE (Cat# 565248); CD206–BV421 (Cat# 566281). Isotype controls (all 1:20): FITC isotype (Cat# 554679); PE isotype (Cat# 554680); APC isotype (Cat# 555576); BV421 isotype (Cat# 562438).
Validation	All antibodies are commercially available and validated by the manufacturer for flow cytometry in human samples, with validation data and citations provided on the suppliers' product pages.

Eukaryotic cell lines

Policy information about cell lines and Sex and Gender in Research

Cell line source(s)	Human iPSC-derived macrophages (iPSDM) were generated from the KOLF2-C1 AAVS1:EGFP human induced pluripotent stem cell line. This cell line was a gift from the Skarnes Lab (The Jackson Laboratory, CT, USA) and expresses EGFP under the control of the CAGG promoter inserted at the AAVS1 safe harbor locus. The sex of the donor cell line is male.
Authentication	The KOLF2-C1 AAVS1 cell line was authenticated by short tandem repeat (STR) profiling prior to use in experiments.
Mycoplasma contamination	All cell lines were routinely tested and confirmed negative for mycoplasma contamination.
Commonly misidentified lines (See ICLAC register)	No commonly misidentified cell lines (per ICLAC register) were used in this study.

Animals and other research organisms

Policy information about studies involving animals; ARRIVE guidelines recommended for reporting animal research, and Sex and Gender in Research

Laboratory animals	C3HeB/FeJ female mice (Mus musculus), aged 6–8 weeks at the time of infection, were used in this study. Mice were bred and maintained under specific pathogen-free conditions at the Francis Crick Institute.
Wild animals	This study did not involve wild animals.
Reporting on sex	Only female mice were used in this study and as such, sex-based analysis was not performed.
Field-collected samples	This study did not involve field-collected samples.
Ethics oversight	All animal procedures were approved by the Francis Crick Institute Animal Welfare and Ethical Review Body (AWERB) and conducted under UK Home Office project licence P4D8F6075, in accordance with the Animals (Scientific Procedures) Act 1986.

Note that full information on the approval of the study protocol must also be provided in the manuscript.

Plants

Seed stocks	*Report on the source of all seed stocks or other plant material used. If applicable, state the seed stock centre and catalogue number. If plant specimens were collected from the field, describe the collection location, date and sampling procedures.*
Novel plant genotypes	*Describe the methods by which all novel plant genotypes were produced. This includes those generated by transgenic approaches, gene editing, chemical/radiation-based mutagenesis and hybridization. For transgenic lines, describe the transformation method, the number of independent lines analyzed and the generation upon which experiments were performed. For gene-edited lines, describe the editor used, the endogenous sequence targeted for editing, the targeting guide RNA sequence (if applicable) and how the editor was applied.*
Authentication	*Describe any authentication procedures for each seed stock used or novel genotype generated. Describe any experiments used to assess the effect of a mutation and, where applicable, how potential secondary effects (e.g. second site T-DNA insertions, mosiacism, off-target gene editing) were examined.*

Flow Cytometry

Plots

Confirm that:

- [x] The axis labels state the marker and fluorochrome used (e.g. CD4-FITC).
- [x] The axis scales are clearly visible. Include numbers along axes only for bottom left plot of group (a 'group' is an analysis of identical markers).
- [ ] All plots are contour plots with outliers or pseudocolor plots.
- [x] A numerical value for number of cells or percentage (with statistics) is provided.

Methodology

Sample preparation	Infected iPSC-derived macrophages were lysed at 2h, 24h, and 96h post-infection using 0.05% Triton X-100. Released M. tuberculosis were fixed overnight in 4% PFA at 4 °C. Fixed bacteria were washed and resuspended in PBS + 1% BSA prior to acquisition.
Instrument	Samples were analyzed using a BD LSRFortessa™ Cell Analyzer (BD Biosciences).
Software	Data were acquired using BD FACSDiva software v8.0.1 and analysed using FlowJo v10.10.0 (FlowJo, LLC). Flow cytometry data were analyzed using FlowJo v10.10.0 (FlowJo, LLC). Gating and ratio quantification were performed within FlowJo using standard channel ratio tools.
Cell population abundance	At least 10,000 single-bacterium events were recorded per condition. Populations were compared using the Timer green-to-red ratio (G/R ratio) as a proxy for bacterial replication rate. Quantitative shifts in population ratios are described in Fig. 4B–E and Supplementary Fig. S7–S8.
Gating strategy	Gating was performed based on FSC/SSC to exclude debris and aggregates, followed by single-bacterium gating using red vs green fluorescence. “High G/R” and “low G/R” populations were defined relative to the untreated control gate at 72h post-infection.

- [x] Tick this box to confirm that a figure exemplifying the gating strategy is provided in the Supplementary Information.
