# Supplementary material for: Fast-growing intracellular Mycobacterium tuberculosis populations evade antibiotic treatment
Source: Nat Commun. 2026 Jul 28;17:9164. doi: 10.1038/s41467-026-75915-8 (PMC13522540; doi:10.1038/s41467-026-75915-8)
Supplement: Supplementary file 4 [file 41467_2026_75915_MOESM7_ESM.pdf]

nature portfolio

Peer Review File

Fast-growing intracellular Mycobacterium tuberculosis populations evade antibiotic treatment

Corresponding Author: Dr Maximiliano Gutierrez

This manuscript has been previously reviewed at another journal. This document only contains information relating to versions considered at Nature Communications.

This file contains all reviewer reports in order by version, followed by all author rebuttals in order by version.

Version 0:

Reviewer comments:

Reviewer #1

(Remarks to the Author)
The authors have adequately addressed all my previous comments and questions.

(Remarks on code availability)

Reviewer #2

(Remarks to the Author)
The authors have largely answered our concerns in their point-by-point rebuttal and the findings and tools developed in this study are clearly worth publishing. The introduction has been extended, including some information about the deltaRD1 mutant to underscore its relevance for this study. An effort has been made in the discussion to distinguish conclusions of the study and speculations. In the following, please find a few points to follow up on the authors' responses, and suggest small edits, mostly about minor points. The in cellulo data support the conclusions drawn about the correlation between antibiotic treatment and the high green-to-red ratio cells (faster growth rates) … as long as the variance is not explained by the day tot day / technical variability (see last major point).
However, there is still one point to clarify about the data presented in Fig 3 and their analysis. I had a hard time to relate conclusions to the data presented in the figure, and what I understood is different in a number of instances from the author's conclusions. I believe this chapter should be carefully rewritten for clarification (make sure no one understands as I do if I'm wrong, correct if you adopt the analyses presented below). Here are the main points of divergence:
- The data presented on Timer fluorescence in Figure 3 are really difficult to relate quantitatively (in terms of the proportions of the different – slow, medium, fast - populations) to the result presented in figure 2.
- Green-to-red ratios should be the principal element to present, and even more relevant is the tail of high green-to-red ratio Mtb cells. To that respect, former figure 3E was more readable (maybe with a logarithmic y-scale to highlight the tail of high green-to-red ratio). The analysis of marginal distributions needs to come after this major point and is important to analyze the origin of the shifts in ratios.
- L.218, the reference to Fig. S7A-D does not properly match the message of the sentence (chloramphenicol treatment effect on Timer fluorescence levels). Probably S7A, B would be better suited.
- Please clarify the validation purposes (controls) of the data presented in Fig. S7: those data are apparently acquired on Mtb cells grown in bulk. However, this information comes after (l.221 "Then, using an infection workflow", and l.229).
- L.224-226: the linear relationship between the green and red fluorescence levels reflects a highly similar green-to-red ratio for cells irrespective of the protein production level, over 2 orders of magnitude of fluorescence levels. A change in this ratio should be associated with a change of slope. If the ratio is smaller (for the bulk cells), the slope is smaller and the deviation between the 2 trends gets larger as fluorescence levels increase. High intensity cells getting out of the control gate after treatment is most likely due to the downshift of the ratio in bulk cells rather than a difference of antibiotic effect on cells correlated with their production level of Timer.
- l.226-229: data presented in Fig 3C are used to highlight the presence of a fraction of cells with high green-to-red ratio, interpreted as fast growing subpopulation. However, this is not shown by the shift in regression lines according to the

antibiotic treatments. The shift illustrates the reduction of the green-to-red ratio of the bulk cells.
- l.229-232: the data presented in Fig. S7C-D do not directly illustrate a change in green-to-red ratio. It is actually quite hard to tell whether a fraction of cells with high green-to-red ratio is still present or not on the scatter plots. All panels present some densities above the control gate…
- By the way, in Fig. S7D lower left panel, the chloramphenicol treatment effect does not seem to result in the same decrease in both fluorescence levels (Fig. S7E left panel). In fact, in this Fig. S7D panel, both fluorescence levels seem to have increased compared to the control (Fig. S7D upper left panel).
- In the same vein, the INH EC99 (and to a lower extent the PZA EC99 treatment as well) results in a lower green-to-red ratio of the bulk cells through a higher increase in red than green fluorescence, but with an increase of both fluorescence levels for the vast majority of cells compared to control cells. This is visible in both Fig. 3B and Fig. S7D, but different from Fig. S7E, where all 4 conditions are different from the Fig. S7D lower panels. Please provide an explanation for these discrepancies.
- Fig. 3D is confusing, mis-referenced (not conveying any information about the message in the text where it is referenced) and probably misleading. The analysis of the proportion of cells remaining in the control gate after treatment is more related to the change in fluorescence levels of the majority of cells than to the small fraction of cells with high green-to red ratio. The graph in Fig. 3D is indicative of the downshift of the green-to-red ration for the vast majority of cells and says nothing about the high green-to-red ratio cells.
- Conclusions (l.240-245) are valid and largely supported by the data, but not clearly illustrated. The conclusion is about the "presence of a tail of high GR ratio bacteria…" This tail is only illustrated with the green dots in Fig. 3C and this representation says nothing about the representativity of these sets of points. Despite its limitations, the type of representation of Fig. 2D served better this purpose.

About the new Figure 4: while the data look compelling and may justify conclusions on the correlation between bacterial loads and the replicative state of the cell that escape antibiotic treatments (through the green-to-red ratio proxy).
- Please highlight what is deemed to be a bacterial cell on microscopy images. The bright regions in both green and red channels that are more similar to an organelle than to a Mtb cell are worrying: How are they taken into consideration in the analysis? This is all the more relevant that the images presented for the PZA treatment, which appear to favor high green-to-red ratio / fast replicating cells, does not appear to present fluorescent regions with a bacterial shape.
- Please, speculate on the technical or biological reasons that may explain how in these in cellulo experiments the treatment are driving the green-to-red ratio of the whole bacterial population substantially higher (PZA treatment) or do not affect the ratio (RIF treatment) while through flow cytometry analysis the ratio of the bulk cells was substantially lowered? Are the authors analyzing a subset of the bacterial cell population through microscopy (undetectable bacterial cells in these tissue slices if Timer expression is too low?).
- The correlation analysis appears strong. But given the unusual distribution of CFU/mL and green-to-red ratios (Fig 4E, F – VEHICLE and RIF), it appears important to verify that the variance in these treatment conditions is of biological origin and not technically driven. Which part of the variance is explained by the technical repeats (day to day variance)? This variance is drives the correlation coefficients in Fig. 4G.

Minor points

- Concerning the KDE drawbacks, I understand it is a problem to display negative tails for growth rates distribution when no data point support such values. I am not pushing for the use of kde and given the message of the figure. However, the bandwidth parameter of the kernel density estimator can be changed to avoid over smoothing the data, hence avoiding long tails way beyond extrema of the raw data set. See for example the Sheather and Jones method (https://dx.doi.org/10.1111/j.2517-6161.1991.tb01857.x)
As mentioned in the rebuttal, the point is indeed to showcase the shape of the distributions and the fast-replicating tails. (p.8).
- Concerning the introduction of the deltaRD1 mutant (l.69-70): consider adding " -- i.e., largely contained within the phagosome --" to clarify?
- The use of GMM is indeed a good idea to establish the non-normality of the distributions. It can be used to roughly estimate the contributions of each component (fast/slow growth component). However, it is very difficult to state that the distribution is multimodal. Please, do not conclude something else than the initial assumption of the statistical test, which in this case is: "Are the data better described with 1 or 2 (or more) Gaussian components?". This test says nothing about the possibility to describe the data with one or more heavy tail distribution which would be equally qualified as a Gaussian distribution to model components of a multimodal distribution of generation times. Again, the point is not to remove the GMM fits, which are useful to model the different subpopulations you choose as categories.
- l.156 Consider modifying "The distribution median IS INCLUDED in the canonical 20-25h..." as included seems more suitable than "encompasses" based on the meaning of the rest of the sentence.

- Fig S1B, schematic (right panel) - Isn't it supposed to be the same 'cell 2' on both sides of the arrow if it corresponds to Mtb uptake by a cell?
- Fig S4 is missing?

(Remarks on code availability)

Reviewer #3

(Remarks to the Author)
This is a revised version of a previously submitted manuscript.
The study uses a high-throughput, live-cell imaging approach to measure Mtb replication within human macrophages. The authors identify the presence of fast-growing intracellular Mtb populations that are enriched upon antibiotic treatment. The presence of these subpopulations was also demonstrated during treatment of infected mice.

The authors have responded to the comments from the earlier review and provided additional datasets (rebuttal figures) as well as new experiments (mice infection with Mtb-timer) that supports their observations. Overall, the claims are better supported now and reads well.

Specific comments.
Fig. 3B, C and Fig. S7 – The authors when presenting the data with the Timer protein keep referring to G/R ratios in the text. But most of the plots in the Figures show xy plots of Green vs Red. It might be useful to provide the G/R ratios as a supplementary table, similar to values shown in Fig. 4F.
Ln 55 – Reference #24 is likely cited wrongly
Ln 61 – Reference #30 does not involve intracellular bacteria
Ln 96 – Fig (1H) shouldn't this be 75 h and not 35 h, as stated
Ln 179 – Fig. S4 missing in the supplementary file.
Ln 412 – typo- "Legions" should be "Lesions"

FIgure legends for the supplementary Figures were missing.

(Remarks on code availability)

Open Access This Peer Review File is licensed under a Creative Commons Attribution 4.0 International License, which permits use, sharing, adaptation, distribution and reproduction in any medium or format, as long as you give appropriate credit to the original author(s) and the source, provide a link to the Creative Commons license, and indicate if changes were made.
In cases where reviewers are anonymous, credit should be given to 'Anonymous Referee' and the source.
The images or other third party material in this Peer Review File are included in the article's Creative Commons license, unless indicated otherwise in a credit line to the material. If material is not included in the article's Creative Commons license and your intended use is not permitted by statutory regulation or exceeds the permitted use, you will need to obtain permission directly from the copyright holder.
To view a copy of this license, visit https://creativecommons.org/licenses/by/4.0/

Reviewer #2 (Remarks to the Author):
The authors have largely answered our concerns in their point-by-point rebuttal and the findings and tools developed in this study are clearly worth publishing.
The introduction has been extended, including some information about the deltaRD1 mutant to underscore its relevance for this study.
An effort has been made in the discussion to distinguish conclusions of the study and speculations.
In the following, please find a few points to follow up on the authors' responses, and suggest small edits, mostly about minor points.

The *in cellulo* data support the conclusions drawn about the correlation between antibiotic treatment and the high green-to-red ratio cells (faster growth rates) … as long as the variance is not explained by the day to day / technical variability (see last major point).

However, there is still one point to clarify about the data presented in Fig 3 and their analysis. I had a hard time to relate conclusions to the data presented in the figure, and what I understood is different in a number of instances from the author's conclusions. I believe this chapter should be carefully rewritten for clarification (make sure no one understands as I do if I'm wrong, correct if you adopt the analyses presented below).

Here are the main points of divergence:
- The data presented on Timer fluorescence in Figure 3 are really difficult to relate quantitatively (in terms of the proportions of the different – slow, medium, fast - populations) to the result presented in figure 2.

> We agree with the reviewer's comment. Considering the intrinsic maturation properties of Mtb-Timer, the reporter only serves as a proxy of the bacterial replication rate, and we do not intend to quantitatively link the phenomenon reported in Figure 2 and 3. We have now added clarification at line 217-219 to highlight the data presented in Figure 2 and 3 shall not be interpreted as quantitatively linked.

- Green-to-red ratios should be the principal element to present, and even more relevant is the tail of high green-to-red ratio Mtb cells. To that respect, former figure 3E was more readable (maybe with a logarithmic y-scale to highlight the tail of high green-to-red ratio). The analysis of marginal distributions needs to come after this major point and is important to analyze the origin of the shifts in ratios.

> We agree with the reviewer that the G/R ratio distribution should be a primary element in Figure 3. We have reinstated the former Figure 3E, replotted with a logarithmic y-scale to emphasise the tail of high G/R ratio bacteria that persists across all antibiotic conditions.

- L.218, the reference to Fig. S7A-D does not properly match the message of the sentence (chloramphenicol treatment effect on Timer fluorescence levels). Probably S7A, B would be better suited.

We thank the reviewer for this observation. Following restructuring of Supplementary Figure S7, the chloramphenicol treatment data is now presented in panel S7B and the in-text reference has been updated accordingly.

- Please clarify the validation purposes (controls) of the data presented in Fig. S7: those data are apparently acquired on Mtb cells grown in bulk. However, this information comes after (l.221 “Then, using an infection workflow”, and l.229).

We thank the reviewer for this observation. To clarify the validation context, supplementary figure S7 has been restructured to present exclusively *in vitro* data, with clear icon-based keys to denote the *in vitro* culture context. The *in cellulo* data is presented separately in a new supplementary figure S8, also with accompanying key icon.

- L.224-226: the linear relationship between the green and red fluorescence levels reflects a highly similar green-to-red ratio for cells irrespective of the protein production level, over 2 orders of magnitude of fluorescence levels. A change in this ratio should be associated with a change of slope. If the ratio is smaller (for the bulk cells), the slope is smaller and the deviation between the 2 trends gets larger as fluorescence levels increase. High intensity cells getting out of the control gate after treatment is most likely due to the downshift of the ratio in bulk cells rather than a difference of antibiotic effect on cells correlated with their production level of Timer.

We agree with the reviewer’s interpretation. This is consistent with antibiotics affecting both bacterial replication and protein expression simultaneously in the intracellular context, as described in Campo-Perez et al. (ref: 41)

- l.226-229: data presented in Fig 3C are used to highlight the presence of a fraction of cells with high green-to-red ratio, interpreted as fast growing subpopulation. However, this is not shown by the shift in regression lines according to the antibiotic treatments. The shift illustrates the reduction of the green-to-red ratio of the bulk cells.

We agree with the reviewer. We have clarified the interpretation of Fig. 3C in the revised text at lines 230 onwards.

- l.229-232: the data presented in Fig. S7C-D do not directly illustrate a change in green-to-red ratio. It is actually quite hard to tell whether a fraction of cells with high green-to-red ratio is still present or not on the scatter plots. All panels present some densities above the control gate…

We agree with the reviewer. Fig. S8B (previously Fig. S7D) illustrates the concentration dependent reduction in the proportion of bacteria within the control gate, reflecting the bulk downshift in G/R ratio rather than directly demonstrating the presence of high G/R ratio cells. The persistence of the fast-growing subpopulation is instead illustrated in Fig. 3C as described above. We have updated the results text at line 226 to reflect this.

- By the way, in Fig. S7D lower left panel, the chloramphenicol treatment effect does not seem to result in the same decrease in both fluorescence levels (Fig. S7E left panel). In fact, in this Fig. S7D panel, both fluorescence levels seem to have increased compared to the control (Fig. S7D upper left panel).

> We thank the reviewer for this observation. The apparent discrepancy arises because the panels referred to as Fig. S7D and S7E in the previous submission represented *in cellulo* and *in vitro* data respectively. To eliminate this source of confusion, we restructured the supplementary figures so that *in vitro* and *in cellulo* data are presented in separate figures (S7 and S8 respectively).

- In the same vein, the INH EC99 (and to a lower extent the PZA EC99 treatment as well) results in a lower green-to-red ratio of the bulk cells through a higher increase in red than green fluorescence, but with an increase of both fluorescence levels for the vast majority of cells compared to control cells. This is visible in both Fig. 3B and Fig. S7D, but different from Fig. S7E, where all 4 conditions are different from the Fig. S7D lower panels. Please provide an explanation for these discrepancies.

> We thank the reviewer for this observation. As noted above, the discrepancy between Fig. 3B/S7D and S7E in the previous submission reflects the fundamental difference between *in cellulo* and *in vitro*. This has been addressed with the new supplementary Fig. S7 and S8.

- Fig. 3D is confusing, mis-referenced (not conveying any information about the message in the text where it is referenced) and probably misleading. The analysis of the proportion of cells remaining in the control gate after treatment is more related to the change in fluorescence levels of the majority of cells than to the small fraction of cells with high green-to red ratio. The graph in Fig. 3D is indicative of the downshift of the green-to-red ration for the vast majority of cells and says nothing about the high green-to-red ratio cells.

> We agree with the reviewer that Fig. 3D was mis-referenced in the previous version. We have now re-referenced it to correctly convey that a consistent proportion of intracellular Mtb remains within the control gate across all treatment conditions, indicating that a subpopulation exists that is unlikely to be directly influenced by antibiotic pressure.

- Conclusions (l.240-245) are valid and largely supported by the data, but not clearly illustrated. The conclusion is about the “presence of a tail of high GR ratio bacteria…” This tail is only illustrated with the green dots in Fig. 3C and this representation says nothing about the representativity of these sets of points. Despite its limitations, the type of representation of Fig. 2D served better this purpose.

> We agree that the Fig. 3C alone is insufficient to convey the representativity of the high G/R ratio subpopulation. The reinstated Fig. 3E addresses this

directly, displaying the fully G/R ratio distribution per condition on a log-y scale, ensuring the high-ratio tail is clearly visible.

About the new Figure 4:
- while the data look compelling and may justify conclusions on the correlation between bacterial loads and the replicative state of the cell that escape antibiotic treatments (through the green-to-red ratio proxy).
- Please highlight what is deemed to be a bacterial cell on microscopy images. The bright regions in both green and red channels that are more similar to an organelle than to a Mtb cell are worrying: How are they taken into consideration in the analysis? This is all the more relevant that the images presented for the PZA treatment, which appear to favor high green-to-red ratio / fast replicating cells, does not appear to present fluorescent regions with a bacterial shape.

We thank the reviewer for raising this point, which required clarification in the manuscript. Bacterial segmentation in tissue sections was performed semi-automatically using the brush tool in QuPath (lines 612-617, Materials and Methods), with the outcome of this approach displayed in Supplementary Figure 9. To clarify which structures are quantified in the main figures, we have added annotations to the insets of Fig. 4B–D, with bacteria delineated by yellow dotted lines. To address the reviewer's concern more specifically, we have also annotated elements excluded from quantification in magenta dotted lines, namely erythrocytes, identifiable by their characteristic morphology and bright signal in both green and red channels. Using this semi-automated segmentation approach, we are confident that the green-to-red ratios reported reflect bacterial signal specifically.

- Please, speculate on the technical or biological reasons that may explain how in these in cellulo experiments the treatment are driving the green-to-red ratio of the whole bacterial population substantially higher (PZA treatment) or do not affect the ratio (RIF treatment) while through flow cytometry analysis the ratio of the bulk cells was substantially lowered? Are the authors analyzing a subset of the bacterial cell population through microscopy (undetectable bacterial cells in these tissue slices if Timer expression is too low?).

We thank the reviewer for this comment. Out in cellulo data shows that EC99 treatment enriches the fast-growing Mtb subpopulation, while our *in vivo* data shows that treatment failure is associated with a higher or unchanged G/R ratio. We speculate that the key difference between these observations lies in the kinetics of antibiotic action: the fast-growing subpopulation represents a minor fraction *in cellulo* at 96h post-infection, but under prolonged antibiotic pressure *in vivo*, slow-growing bacteria are progressively cleared, leaving fast-growing, antibiotic tolerant bacteria increasingly dominant in treatment-failure outcomes. This speculation has been added to the discussion (lines 329-336).

- The correlation analysis appears strong. But given the unusual distribution of CFU/mL and green-to-red ratios (Fig 4E, F – VEHICLE and RIF), it appears important to verify that the variance in these treatment conditions is of biological origin and not

technically driven. Which part of the variance is explained by the technical repeats (day to day variance)? This variance is drives the correlation coefficients in Fig. 4G.

We thank the reviewer for raising this important point. We wish to clarify that Figure 4 describes i*n vivo* mouse infection experiments, where each mouse represents an independent biological unit (Lazic et al. 2018, doi:10.1371/journal.pbio.2005282), and the variance observed in Fig. 4E–F therefore primarily reflects inter-individual biological variability rather than technical replication.

To directly address the reviewer's concern, we performed a series of statistical analyses to access the contribution of technical variance between the two independent infection experiments. First, unpaired t-tests comparing lung CFU and mean G/R ratios between infection batches within each treatment group revealed no significant differences for vehicle or PZA (all $p<0.05$), supporting a biological origin of variance in these conditions. For RIF, significant between batch differences were observed (CFU: $p=0.002$; G/R ratios: $p<0.001$), prompting further investigation.

To determine whether these batch differences reflect a systematic technical confound or a condition-specific biological phenomenon, we performed a two-way ANOVA with condition, batch and their interaction as predictors. For lung CFU, the condition-by-batch interaction was non-significant ($F=0.22$, $p=0.801$), indicating that any batch effect on bacterial burden is consistent across all treatment groups and therefore unlikely to represent a systematic technical artefact. For mean G/R ratio, the interaction term was close to statistically significant ($F=3.48$, $p=0.050$), suggesting that the batch effect on timer expression differs by condition. This perspective is consistent with a condition-specific biological phenomenon rather than a global technical confounding effect.

This interpretation is further supported by the Vehicle group, which displays a similar stratification of G/R ratios into two distinct subpopulations across both infection experiments. This is a pattern that mirrors the distribution observed in RIF-treated mice. Since vehicle mice received no antibiotic treatment and showed no significant between-experiment differences, (CFU: $p=0.473$; G/R ratio: $p=0.581$), this stratification cannot be attributed to technical artefacts, demonstrating that biological heterogeneity of this magnitude exists independently of infection batch.

We therefore propose that the between-experiment variance in RIF-treated mice reflects the stochastic nature of antibiotic treatment failure *in vivo*. The rare enrichment of fast-growing, antibiotic-tolerant Mtb subpopulations in a subset of hosts, directly consistent with the *in cellulo* phenotype described in previously. The full statistical results are provided in the following table:

Table A — Per-condition unpaired t-tests (batch 1 vs batch 2)

Condition	Metric	Batch 1 mean	Batch 2 mean	t	p

Vehicle	Lung CFU (log10)	6.327	6.688	-0.758	0.473
Vehicle	Mean G/R ratio	1.364	0.989	0.579	0.581
RIF	Lung CFU (log10)	4.668	5.602	-4.887	0.002
RIF	Mean G/R ratio	0.489	1.768	-9.608	<0.001
PZA	Lung CFU (log10)	4.232	4.840	-0.675	0.521
PZA	Mean G/R ratio	1.617	3.015	-2.231	0.061

Table B — Two-way ANOVA: condition × batch interaction

Metric	F (interaction)	p (interaction)
Lung CFU (log10)	0.22	0.801
Mean G/R ratio	3.48	0.050

Table C — Partial correlations controlling for batch

Condition	Raw r	Raw p	Partial r	Partial p	n
Vehicle	0.74	0.023	0.85	0.004	9
RIF	0.86	0.003	0.09	0.815	9
PZA	0.71	0.034	0.74	0.023	9

Minor points

- Concerning the KDE drawbacks, I understand it is a problem to display negative tails for growth rates distribution when no data point support such values. I am not pushing for the use of kde and given the message of the figure. However, the bandwidth parameter of the kernel density estimator can be changed to avoid over smoothing the data, hence avoiding long tails way beyond extrema of the raw data set. See for example the Sheather and Jones method (https://dx.doi.org/10.1111/j.2517-6161.1991.tb01857.x)
As mentioned in the rebuttal, the point is indeed to showcase the shape of the distributions and the fast-replicating tails. (p.8).

We thank the reviewer for this clarification. As noted in our previous rebuttal, we elected to display the doubling time distributions as violin plots rather than KDE, as this representation more faithfully captures the underlying data without extrapolating beyond the observed range while still conveying the shape of the distribution and the fast-replicating tail. We have retained this approach in the revised manuscript.

- Concerning the introduction of the deltaRD1 mutant (l.69-70): consider adding " --i.e., largely contained within the phagosome --" to clarify?

We thank the reviewer for this suggestion and have now included this point at line 70-71.

- The use of GMM is indeed a good idea to establish the non-normality of the distributions. It can be used to roughly estimate the contributions of each component

(fast/slow growth component). However, it is very difficult to state that the distribution is multimodal. Please, do not conclude something else than the initial assumption of the statistical test, which in this case is: “Are the data better described with 1 or 2 (or more) Gaussian components?”. This test says nothing about the possibility to describe the data with one or more heavy tail distribution which would be equally qualified as a Gaussian distribution to model components of a multimodal distribution of generation times. Again, the point is not to remove the GMM fits, which are useful to model the different subpopulations you choose as categories.

> We thank the reviewer for this important clarification. We agree that GMM selection via BIC addresses only whether 1 or 2+ Gaussian components better describe the data, and does not exclude alternative explanations such as a heavy-tailed unimodal distribution. We have revised the relevant text to reflect this (main text 162-165 and supplementary materials lines 86-89), replacing conclusions of multimodality with the more conservative statement that the data are better described by a 2-component Gaussian mixture. The GMM fits are retained as a practical framework for categorising bacterial subpopulations, without implying they constitute proof of a multimodal underlying distribution.

- l.156 Consider modifying "The distribution median IS INCLUDED in the canonical 20-25h..." as included seems more suitable than "encompasses" based on the meaning of the rest of the sentence.

> We thank the reviewer for this and have updated line 159 to reflect this suggestion.

- Fig S1B, schematic (right panel) - Isn't it supposed to be the same 'cell 2' on both sides of the arrow if it corresponds to Mtb uptake by a cell?
- Fig S4 is missing? ->

> We thank the reviewer for identifying these issues. Regarding Fig. S1B, the reviewer is correct. The schematic has now been updated so that the same 'cell 2' appears on both sides of the arrow, accurately representing Mtb uptake by a single cell. Regarding Fig. S4, we apologise for the omission in the submitted supplementary file. This figure has been included in the revised submission.

Reviewer #3 (Remarks to the Author):
This is a revised version of a previously submitted manuscript. The study uses a high-throughput, live-cell imaging approach to measure Mtb replication within human macrophages. The authors identify the presence of fast-growing intracellular Mtb populations that are enriched upon antibiotic treatment. The presence of these subpopulations was also demonstrated during treatment of infected mice. The authors have responded to the comments from the earlier review and provided additional datasets (rebuttal figures) as well as new experiments (mice infection with Mtb-timer) that supports their observations. Overall, the claims are better supported now and reads well.
Specific comments.

Fig. 3B, C and Fig. S7 – The authors when presenting the data with the Timer protein keep referring to G/R ratios in the text. But most of the plots in the Figures show xy plots of Green vs Red. It might be useful to provide the G/R ratios as a supplementary table, similar to values shown in Fig. 4F.

We thank the reviewer for this suggestion. To address the difficulty in extracting quantitative G/R ratio information from the xy scatter plots in Fig. 3B and Fig. S8 (formerly Fig. S7), we have reinstated the G/R ratio distribution histogram (formerly Fig. 3E) as a primary panel in the revised Fig. 3. This representation directly displays the single-cell G/R ratio distributions per condition, including the tail of high-ratio bacteria, and is more informative than a summary table for data of this scale (n > 8,000 cells per condition). Notably, the high G/R ratio subpopulation of interest would be obscured in tabulated summary statistics whilst Fig. 3E preserves the full distributional shape and makes the outlier tail directly visible to the reader.

Ln 55 – Reference #24 is likely cited wrongly.

We thank the reviewer for noticing this errant citation and have removed it.

Ln 61 – Reference #30 does not involve intracellular bacteria.

We thank the reviewer for noticing this and have amended the citation.

Ln 96 – Fig (1H) shouldn't this be 75 h and not 35 h, as stated?

We thank the reviewer for this astute observation and have added a clarifying clause that explains that this is the minimum track length rather than the total time lapse duration.

Ln 179 – Fig. S4 missing in the supplementary file.

We thank the reviewer for noticing this and Fig. S4 has been included in the revised supplementary file.

Ln 412 – Typo: "Legions" should be "Lesions".

We thank the reviewer for spotting this typographical error. "Legions" has been corrected to "Lesions" at Ln 412 (now 428).

Figure legends for the supplementary figures were missing.

Figure legends for all supplementary figures have been added to the revised supplementary file.
